# A novel multi-stage distributed authentication scheme for smart meter communication

**DOI:** 10.7717/peerj-cs.643

**Published:** 2021-07-15

**Authors:** Manjunath Hegde, Adnan Anwar, Karunakar Kotegar, Zubair Baig, Robin Doss

**Affiliations:** 1 Department of Computer Applications, Manipal Institute of Technology, Manipal Academy of Higher Education, Manipal, India; 2 School of IT, Centre for Cyber Security Research and Innovation (CSRI), Deakin University, Geelong, VIC, Australia

**Keywords:** Smart meter, Authentication, Security, Smart grid, Cybersecurity

## Abstract

Smart meters have ensured effective end-user energy consumption data management and helping the power companies towards network operation efficiency. However, recent studies highlighted that cyber adversaries may launch attacks on smart meters that can cause data availability, integrity, and confidentiality issues both at the consumer side or at a network operator’s end. Therefore, research on smart meter data security has been attributed as one of the top priorities to ensure the safety and reliability of the critical energy system infrastructure. Authentication is one of the basic building blocks of any secure system. Numerous authentication schemes have been proposed for the smart grid, but most of these methods are applicable for two party communication. In this article, we propose a distributed, dynamic multistage authenticated key agreement scheme for smart meter communication. The proposed scheme provides secure authentication between smart meter, NAN gateway, and SCADA energy center in a distributed manner. Through rigorous cryptanalysis we have proved that the proposed scheme resist replay attack, insider attack, impersonation attack and man-in-the-middle attack. Also, it provides perfect forward secrecy, device anonymity and data confidentiality. The proposed scheme security is formally proved in the CK—model and, using BAN logic, it is proved that the scheme creates a secure session between the communication participants. The proposed scheme is simulated using the AVISPA tool and verified the safety against all active attacks. Further, efficiency analysis of the scheme has been made by considering its computation, communication, and functional costs. The computed results are compared with other related schemes. From these analysis results, it is proved that the proposed scheme is robust and secure when compared to other schemes.

## Introduction

The smart grid is one of the critical infrastructures for any nation. The traditional energy system equipped with advanced sensing, communication and control technologies transforms the decades old power system into a smart grid. While the smart grid provides a wide range of utilities and help the end-users to improve their lifestyle and minimize the cost of energy, new types of cyber-threats have emerged. According to a recent report published by Kaspersky, a noticeable number of cyber incidents were reported in the energy control centres (In H1 2020, ICS computers blocked 32.6% malicious contents) that has raised a major concern (Kaspersky ICS CERT, 2020). For example, a ransomware attack on a large Portuguese energy company was claimed to have stolen around 10TB of sensitive information. As per the report, the major categories of the cyber attacks experienced by the industrial control centre (ICS) of the energy system are worms, spyware and cryptocurrency miners. If not detected, these cyber attacks and new sophisticated threats can cause significant damage to the physcial assets, financial losses, and may even create catastrophic cascading failures. Hence, a good number of research is going on towards the investigation of emerging attacks and threat models (Anwar, Mahmood & Pickering, 2016; Anwar, Mahmood & Pickering, 2017; Anwar, Mahmood & Ahmed, 2015) for the energy system and possible countermeasures (Yang et al., 2014; Anwar, Mahmood & Tari, 2015).

In recent years, smart meters (SM) have been widely deployed to monitor energy usages of the end-user’s consumption data. For example, smart meters have been installed in all residential homes across Victoria, which is one of the major Australian states (DELWP, 2020). SMs have provided the customers insights on their consumption profile and opened the door for data driven informed decision making and control energy at a household level. However, SM data communication is based on wireless medium and making the use of internet technologies. Wireless communication model for SM data transmission may allow malicious attackers to perform various attacks if they do not have a robust security system (Gope & Sikdar, 2018). If the system is compromised by an adversary, the attacker can eavesdrop and modify the communication data. This kind of attack is data privacy and confidentiality attack. Also, the attacker can interrupt the transmitted messages or cause a Denial of Service (DoS) and/or jamming attack against the system that may lead to a data availability attack. Moreover, a new type of attack has emerged where the attacker can inject malicious or false information into the smart meter data payloads and corrupt the original information (Anwar, Mahmood & Tari, 2015). This kind of attack is known as a data integrity attack against smart meters within an advanced metering infrastructures. Hence, attacks on smart meter can impact on the Confidentiality, Integrity and Availability (CIA) triad of an energy systems demand side management. Therefore, the security issues have been highlighted as a major concern of today’s smart grid smart meter communication and data management (Aziz et al., 2019; Ni & Paul, 2019; Anwar, Mahmood & Tari, 2017). Hence, it is necessary to build a powerful security framework for the smart grid system.

Authentication is one of the primary and recommended approaches to address smart grid smart meter communication security issues. In this paper, we have proposed a novel distributed multi-stage authentication scheme for smart grid smart meter communication. There are numerous authentication schemes for smart grid are proposed in recent years. The need for the proposed authentication scheme is that there are drawbacks identified in most of the recently proposed schemes and they fail to achieve security requirements (Zhang et al., 2019). We have listed the potential vulnerabilities which restrict to the providing of secure authentication in the smart grid smart meter communication. Some of the notable drawbacks are: (i) Anonymity and untraceability which is one of the hurdles to achieve the secure authentication. It enables to reveal the identity of the smart meter which holds the partial information of the login message. Also, an unauthorized user may compromise a meter from the physical box and control the home appliances (Kumar et al., 2019). (ii) The smart meter sends unit consumption data periodically: 15/30/60 minutes. The participants involved in the smart grid smart meter communication are connected via the network Kumar et al. (2018). Since the communication has been done through the open channel, the adversary can intercept the network to leak, modify or delete data (Wang & Lu, 2013). Communication interception may leads towards man in middle attack, replay attack and so on.

In smart energy networks, power transmission and distribution system is separated from the data communication network (Fouda et al., 2011). In power transmission and distribution system, electricity is delivered from the power plant to end-users. In this paper, we described the network from the data communications point of view. The detailed system design is illustrated in the “System Model”. Traditionally, the Supervisory Control and Data Acquisition (SCADA) system has been used to monitor and control the power grid. The SCADA system is a centralized system, where the substations are connected to a control center. The centralized structure of the SCADA system limits its scalability and makes its applicability only for local monitoring. But, an extended SCADA system makes it to Wide-area monitoring, protection, and control system (Shin, He & Zhang, 2014; Eder-Neuhauser, Zseby & Fabini, 2016). Here, smart grid topology is split into a number of networks (Zhong, Chim & Hui, 2015). The active participants in the communication are the smart meter (SM), home area network (HAN), neighbourhood area network gateway (NANG) and the SCADA control center/server. These participants are organized in a hierarchical structure for smart grid communications. To implement the mutual authentication between the active participants, several schemes depending upon the trusted third party (Li et al., 2013; Li et al., 2017; Wu et al., 2019). Dependency on the third party always a bottleneck for the system efficiency when it is too busy on handling of large incoming requests (Odelu et al., 2017). Also, a trusted third party should be suitable to adopt communication environment (Irshad et al., 2020). The authentication schemes independent of the trusted third party assumed that the network between NANG and SCADA control server is secure (Li et al., 2019; Kumar et al., 2019). Since the communication between NANG and SCADA control center/server will be done in an open channel, there will be the chances for network interception. Therefore, it is necessary to propose a novel distributed authentication scheme, which is independent from the third party and authenticate all active participants involved in the communication. Hence, the contributions of the proposed scheme are:

- We have proposed a novel distributed dynamic multistage authenticated key agreement scheme to achieve the illustrated security problems. The proposed scheme provides secure authentication between SM, NANG, and SCADA control server in a distributed manner, which means the authentication of SM, NANG, and SCADA control server does not depending upon any third party.
- The proposed scheme achieves the authentication in a fully hierarchical manner which is suitable for smart grid smart meter communication architecture.
- The proposed security scheme is formally proved in the CK - model. The scheme is simulated using AVISPA tool to verify the security against all active attacks.
- The proposed scheme’s efficiency analysis has been made, considering its computation, communication, and functional costs. The computed results are compared with the other related schemes.

### Related work

Authentication is a fundamental security solution and it has been extensively studied in various application areas. In smart grid smart meter communication, several authentication schemes have been proposed by numerous researchers. Even after the schemes are developed for specific architecture or application, there may be some similarities observed in terms of authentication factors, cryptographic operation, and message communication (Abbasinezhad-Mood, Ostad-Sharif & Nikooghadam, 2019). In this article, we only reviewed recent and relevant literatures, for the most part, authentication which focuses on the network architecture which includes SM, NAN, and SCADA control server. Wu & Zhou (2011) proposed an anonymous key distribution scheme for the smart grid. This scheme mainly combines the symmetric key and elliptic curve public key techniques. In the scheme, the symmetric key was based on the Needham-Schroeder authentication protocol. Wu & Zhou (2011) claimed that the proposed scheme effectively resists the man-in-the-middle attack and the replay attack. Later, Xia & Wang (2012) analysed Wu & Zhou’s (2011) scheme and identified the scheme is vulnerable to man-in-the-middle attack. To overcome the identified attack, Xia & Wang (2012) proposed a key distribution scheme. Here, the authors used a trusted third party to manage the key distribution for the smart meter and the service provider.

Nicanfar et al. (2013) proposed an efficient mutual authentication scheme. This scheme was developed to authenticate the entities that present outside of the home area network. Nicanfar et al. (2013) assumed that the authenticating participants are a smart meter and authentication server. From this literature, Nicanfar et al. (2013) also proposed a key management protocol based on identity cryptography for secure smart grid communications using the public key infrastructure. Li et al. (2013) proposed a Merkle-Tree-Based authentication scheme for secure smart grid communication. This article is more focused on eliminating message injection, message analysis, message modification, and replay attacks during communication.

Tsai & Lo (2015) reviewed Xia & Wang’s (2012) authentication scheme and identified that Xia & Wang (2012) scheme does not support smart meter anonymity and perfect forward secrecy. To overcome the identified weaknesses, Tsai & Lo’s (2015) proposed a key distribution scheme for smart grid environments. The scheme was proposed including properties of bilinear pairings. Importantly, Tsai & Lo’s (2015) scheme needed a trusted third party to distribute the private keys for smart meters and smart grid during registration. Later, Odelu et al. (2016) reviewed Tsai & Lo’s (2015) scheme and found that the scheme can reveal the smart meter’s secret credentials when the secret key has been revealed. To overcome the identified security weakness, Odelu et al. (2016) proposed a new authenticated key agreement scheme based on bilinear pairings for the smart grid. Like Tsai & Lo (2015) scheme, Odelu et al. (2016) utilized trusted third party to handle the private keys.

Further, Mahmood et al. (2016) proposed a hybrid Diffie-Hellman based lightweight authentication scheme using AES and RSA cryptography. This scheme was focused on achieving authentication between building area networks (BAN) and smart meters. The objective of the scheme was to avoid replay attack during authentication. Wazid et al. (2017) proposed a three-factor user authentication scheme for a renewable energy-based smart grid environment. Here, the objective was to authenticate vehicle user who wants to charge his/her electric vehicle battery. In this scheme, even though there are multiple authorities involved, authentication can be possible only between user and smart meter. Also, Wazid et al.’s (2017) scheme is dependent on trusted authority for smart meter registration.

Mahmood et al. (2018) proposed a lightweight ECC-based authentication scheme for smart grid communication. The aim of the scheme is to authenticate two registered users communicating in the environment. In this scheme, the trusted third party was responsible to generate preliminary parameters like selecting elliptic curve, random base point, one-way hash functions, secret key, and so on. Mahmood et al.’s (2018) scheme was also aimed to eliminate the trade-off between performance and security in smart grid communication where the scheme should provide high security with high performance. Further Abbasinezhad-Mood & Nikooghadam (2018) analyzed Mahmood et al.’s (2018) scheme and identified that the scheme cannot provide the perfect forward secrecy. Also identified that the private key of users and shared session keys can be easily compromised with an adversary. To overcome the identified weaknesses, Abbasinezhad-Mood & Nikooghadam (2018) proposed elliptic curve cryptography based lightweight authentication scheme for smart grid communications. Mahmood et al.’s (2018) scheme was also reviewed by Chen et al. (2019) and identified that the scheme could not provide the perfect forward secrecy and private key privacy. Also, the authors analyzed Abbasinezhad-Mood & Nikooghadam’s (2018) scheme and identified that the scheme is vulnerable to the replay attack. To withstand the identified weaknesses, Chen et al. (2019) proposed a bilinear map pairing-based authentication and key establishment scheme.

Zhang et al. (2019) proposed a lightweight anonymous authentication and key agreement scheme for the smart grid. This scheme allows the smart meter and the service provider to authenticate each other. The authentication scheme does not follow any hierarchical network architecture which minimizes the system applicability. Ma et al. (2019) proposed the eye-movement and iris recognition based portable remote authentication for the smart grid. It was a biometrics-based remote operator authentication scheme that uses the record of eye-movement trajectory and randomly selected iris image for authentication.

Recently, Moghadam et al. (2020) proposed a lightweight key management protocol for secure communication between substation and data center in smart grids. The scheme was based on hash functions and private keys. Irshad et al. (2020) proposed a message authentication scheme for secure communications between HAN gateway and BAN gateway in smart grids. Aghapour et al. (2020) proposed a broadcast authentication scheme for smart grid communications which uses hash functions and private key. Here, the authentication was to be done between the NAN gateway and the smart meter. Sadhukhan et al. (2020) proposed a privacy-preserving authentication scheme for smart-grid communication using elliptic curve cryptography. Here, the scheme can authenticate HAN gateway and BAN gateway in smart grids. This scheme needs a trusted third party to generate credentials, keys, and hash functions. Wu et al. (2021) reviewed Chen et al.’s (2019) scheme and identified the known session-specific temporary information attack where adversary can get the information of random nonce which needed to compute the session key. Also, Wu et al. (2021) identified that Chen et al.’s (2019) scheme is vulnerable to impersonation attack. To overcome identified weaknesses, Wu et al. (2021) proposed an improved pairing-based authentication scheme. The proposed scheme, depended upon a trusted third party to generate secret keys, cyclic groups, and hash functions.

From the above literature study we can observe that (i) most of the authentication schemes have security flaws and their improvements are also show some possible attacks. (ii) Many schemes can perform authentication either between the smart meter and NAN gateway or gateway and SCADA center. (iii) Also, the authentication schemes that implemented to the hierarchical network are dependent upon a trusted third party. Therefore, It is vary much necessary to propose an authentication scheme that can mutually authenticate smart meter, NAN gateway, and SCADA center without involvement of any third party. Hence, we have proposed a novel multistage distributed authentication scheme for smart grid communication.

The rest of the article is assembled as follows. “System Model” presents the system model used to propose the authentication scheme. “Cryptographic Preliminaries” discusses the necessary cryptographic preliminaries to understand the scheme. “The Proposed Scheme” illustrates the proposed multi-stage authentication scheme for smart meter communication. This section includes a detailed explanation of the steps of authentication in each phase. Further, “Cryptanalysis of the Proposed Scheme” presents the cryptanalysis of the proposed scheme. Here, we proved the data confidentiality, sensitivity and the security provided in the proposed scheme. The formal security verification of the proposed scheme based on the CK adversary model is illustrated in “Formal Security Proof”, followed by the results of formal security verification using AVISPA are presented in “Result of Formal Security Verification Using Avispa Tool”. “Efficiency Analysis” discusses the proposed scheme’s efficiency analysis by comparing result computation cost (“Computation Cost Analysis”) and communication cost (“Communication Cost”) of the proposed scheme with the other related schemes. This section also gives the comparison result of the essential functionalities (“Functional analysis”) in smart meter communication. Finally, the article depicts the concluding remarks.

## System model

**Figure 1:**
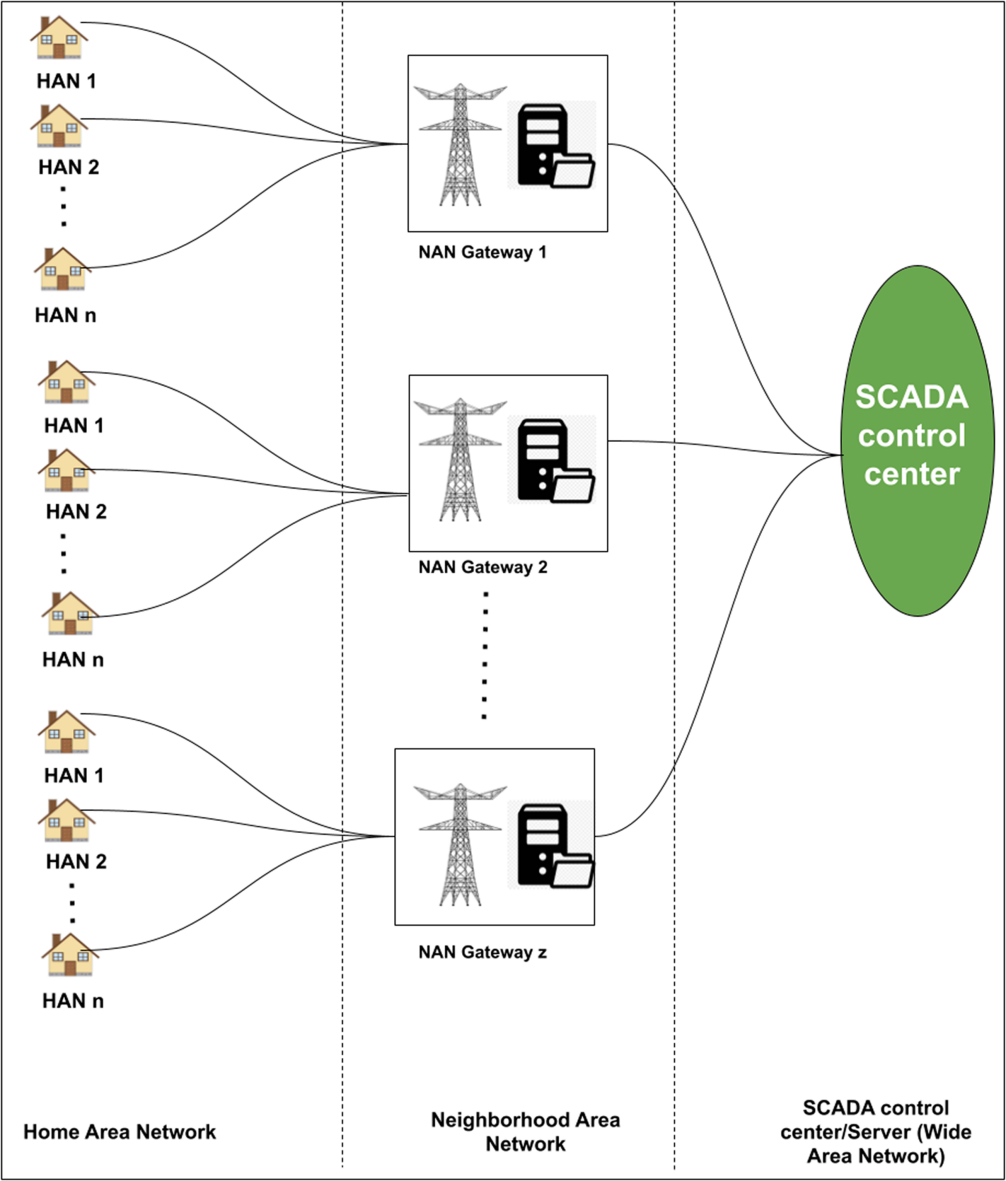
Proposed architecture of the system model.

In this section, we present the system model which involves key components towards a smart meter communication between the consumers and SCADA control centres. As discussed in the “Introduction”, the system model described in the article is from the data communications point of view. The active participants are Smart Meter (SM), Home Area Network (HAN), Neighborhood Area Network Gateway (NANG) and SCADA control center/Server. These participants are well connected in a hierarchical manner. In the considered system architecture, HAN has been considered as the bottom layer. Within a HAN, home appliances are connected to a Smart Meter (SM). The purpose of SM is to collect the aggregated consumption (e.g., dishwasher, electric oven, etc) and generation (e.g., solar PV) profiles of the home devices. The collected data can be transferred to the SCADA control center/Server or smart grid (SG) for data storage and analysis. The communication happens via NANG that has been considered as the middle layer of the system. The complete system architecture is presented in Fig. 1.

## Cryptographic preliminaries

This section discusses the cryptographic preliminaries necessary to understand the proposed scheme. The necessary encryption/decryption operations are done using Elliptic Curve Cryptography (ECC). The security strength of ECC relies heavily on the hardness of solving the elliptic curve discrete logarithm problem (ECDLP). Compared to any other public-key cryptosystems, ECC can provide significant security strength to any communication system with less key size (Hancock, 2001). This property reduces the algorithmic computational cost and makes the protocol more lightweight.

*Elliptic curve cryptography:* The equation of the elliptic curve *E*_*p*_(*a*, *b*) over *F*_*p*_ (*p* > 3 and is a large prime number) is defined as *y*^2^ *mod p* = (*x*^3^ + *ax* + *b*) *mod p* where (4*a*^3^ + 27*b*^2^) *mod p* = 0; *x*, *y*, *a*, *b* ∈ [0, *p* − 1]. Any points (*x*, *y*) ∈ *E*_*p*_(*a*, *b*) are denoted as *E*(*F*_*p*_) = {(*x*, *y*) : *x*, *y* in *F*_*p*_ satisfy ${y^2} = ({x^3} + ax + b)\} \cup {\rm {\cal O}}$ where ${\rm {\cal O}}$ is a point at infinity. The point multiplication is computed by repeated addition as *k.P* = *P* + *P* + *P* + ….. + *P* K times (Koblitz, 1987).

*Elliptic Curve Discrete Logarithm Problem(ECDLP):* An elliptic curve *E* defined over a finite field *GF*(*q*) and two points P, Q ∈ E, it is hard to find an integer x ∈ *Z*_p_* such that Q = xP.

*Elliptic Curve Diffie Hellman (ECDH):* Elliptic Curve Diffie Hellman (ECDH) key exchange is the the classical Diffie-Hellman key exchange which exchanges secret information or secure keys between two parties. The keys are exchanged between A and B as follows.

System selects an elliptic curve *E*_*p*_ over the prime finite field *F*_*p*_ where p is a large prime number and select a point on elliptic curve *P* of order n. The algorithm is as follows.

1. *A* generates a random number *k*_*A*_ in the interval [1, *n* − 1] and performs a scalar multiplication *Q*_*A*_ = *k*_*A*_ × *P* Then, sends *Q*_*A*_ to *B*
2. *B* also generates a random number *k*_*B*_ and computes *Q*_*B*_ = *k*_*B*_ × *P* by scalar multiplication in the same way as described above and sends *Q*_*B*_ to A.
3. After receiving *Q*_*B*_ from *B*, *A* computes *K*_1_ = *k*_*A*_ × *Q*_*B*_. Similarly, *B* receives *Q*_*A*_ from *A* and computes *K*_2_ = *k*_*B*_ × *Q*_*A*_.
4. *A* and *B* shares *K*_1_ and *K*_2_ between them. Thus, two entities exchanges the keys securely.

## The proposed scheme

This section presents the proposed novel distributed dynamic multistage authenticated key agreement scheme for the smart grid. The proposed scheme contains three phases (1) Initialization phase, (2) Registration phase and (3) Authentication phase. The notations used throughout the scheme are presented in Table 1.

**Table 1:** Notations and descriptions.

Notations	Descriptions
*SG*_*i*_	SCADA control center/server
*SM*_*i*_	Smart meter
*E*_*p*_	Elliptic curve of order *p*
*F*_*p*_	Finite field
*NANG*_*i*_	Neighborhood area network gateway
*P*	Point on elliptic curve *E*_*p*_
*s*, *P*_*pub*_	Private/public key pair of *SG*_*i*_ where *P*_*pub*_ = *s.P*
*PW*_*i*_	Password of *U*_*i*_
+	Bitwise XOR operator
||	Concatenation operator
*h*_0_(.), *h*_1_(.), *h*_2_(.)	One-way hash functions
*T*_1_, *T*_2_, *T*_3_, *T*_4_	Timestamps generated by *SM*_*i*_, *NANG*_*i*_, *SG*_*i*_

### Initialization phase

To fully describe the proposed scheme, the process of system initialization is explained first. The initialization has to be done while deploying the system. It includes selecting an elliptic curve, hash functions, public and private keys necessary to perform encryption/decryption, and hashing operation throughout the entire scheme.

*SG*_*i*_ choose an elliptic curve *E*_*p*_ over the finite field *F*_*p*_ where p is a large prime number. *SG*_*i*_ also selects an elliptic curve point P of order n and one way hash functions ${h_0}(.) \to {Z^*},{h_1}(.) \to {Z^*},{h_2}(.) \to {Z^*}$. Further *SG*_*i*_ picks the private key x and computes the public key *P*_*pub*_ = *x.P*. Finally, *SG*_*i*_ publishes the parameters {*E*_*p*_, *P*, *F*_*p*_, *h*_0_(.), *h*_1_(.), *h*_2_(.), *P*_*pub*_}.

### Registration phase

The registration phase includes steps to register participants (e.g., *SM*_*i*_, *NANG*_*i*_, etc.), which take part in the system. This phase includes the registration of Neighborhood Area Network gateway *NANG*_*i*_ and smart meter *SM*_*i*_. The details of *NANG*_*i*_ and *SM*_*i*_ registration have been presented below.

#### NAN gateway registration phase

In the proposed scheme, *NANG*_*i*_ registration is done with a SCADA control center/server. The steps involved in the *NANG*_*i*_ registration have been illustrated as follows:

- *NANG*_*i*_ selects *NID*_*i*_, *b*_*j*_ and computes *A*_*i*_ = *h*_0_(*NID*_*i*_ ||*b*_*j*_)
- Sends the registration request message {*NID*_*i*_, *A*_*i*_} to the *SG*_*i*_.
- *SG*_*i*_ receives the request message, generates the random number e and computes

*m*_*i*_ = *h*_0_(*s* ||*e*),

*V*_*n*_ = *h*_0_(*m*_*i*_ ||*A*_*i*_).*P*_*pub*_ and

*H*_*n*_ = *h*_0_(*V*_*n*_|| *NID*_*i*_)

- *SG*_*i*_ stores *H*_*n*_ into the database and sends *V*_*n*_ to the *NANG*_*i*_. NAN gateway receives *V*_*n*_ and stores {*NID*_*i*_, *b*_*j*_, *V*_*n*_} into its database. The registration phase of NAN gateway has been presented in the Table 2.

**Table 2:** NAN gateway registration phase.

NAN gateway	SCADA control center/server
Selects *NID*_*i*_, *b*_*j*_	
Computes *A*_*i*_ = *h*_0_(*NID*_*i*_|| *b*_*j*_)	
Sends {*NID*_*i*_, *A*_*i*_}	
{*NID_i_,A_i_*} −−−−→	
	Receives message and generates the random number e
	Computes *m*_*i*_ = *h*_0_(*s*|| *e*)
	*V*_*n*_ = *h*_0_(*m*_*i*_ ||*A*_*i*_).*P*_*pub*_ and
	*H*_*n*_ = *h*_0_(*V*_*n*_ ||*NID*_*i*_)
	Stores *H*_*n*_ into the database and sends *V*_*n*_ to the *NANG*_*i*_
	{*V_n_*} ←−−
Receives *V*_*n*_ and stores {*NID*_*i*_, *b*_*j*_, *V*_*n*_}	

#### Smart meter registration phase

The registration of the *SM*_*i*_ is with *NANG*_*i*_. The registration of *SM*_*i*_ has been presented below:

- *SM*_*i*_ selects *MID*_*i*_, *b*_*i*_, computes *A*_*j*_ = *h*_0_(*MID*_*i*_ ||*b*_*i*_) and sends the registration request message {*MID*_*i*_, *A*_*j*_} to the NAN gateway.
- *NANG*_*i*_ receives the request message and computes

*H*_*n*_ = *h*_0_(*V*_*n*_ ||*NID*_*i*_),

*V*_*m*_ = *h*_0_(*H*_*n*_ ||*A*_*j*_).*P*_*pub*_ and

*H*_*m*_ = *h*_0_(*V*_*m*_|| *MID*_*i*_)

- *NANG*_*i*_ stores *H*_*m*_ into the database and sends *V*_*m*_ to the *SM*_*i*_. Smart Meter receives *V*_*m*_ and stores {*MID*_*i*_, *b*_*i*_, *V*_*m*_} into *SM*_*i*_. The Smart meter registration phase has been presented in Table 3.

**Table 3:** Smart meter registration phase.

Smart meter	NAN gateway
*MID*_*i*_, *b*_*i*_	
Computes *A*_*j*_ = *h*_0_(*MID*_*i*_ *b*_*i*_)	
Sends {*MID*_*i*_, *A*_*j*_}	
{*MID_i_,A_j_*} −−−−−→	
	Receives message
	Computes *H*_*n*_ = *h*_0_(*V*_*n*_ ||*NID*_*i*_),
	*V*_*m*_ = *h*_0_(*H*_*n*_ ||*A*_*j*_).*P*_*pub*_ and
	*H*_*m*_ = *h*_0_(*V*_*m*_|| *MID*_*i*_)
	Stores *H*_*m*_ into its database and sends *V*_*m*_ to the *SM*_*i*_.
	{*V_m_*} ←−−
Receives *V*_*m*_ and stores {*MID*_*i*_, *b*_*i*_, *V*_*m*_} into *SM*_*i*_.	

### Authentication phase

The authentication phase is performed between *SM*_*i*_, *NANG*_*i*_, and *SG*_*i*_. Here, *SM*_*i*_ and *SG*_*i*_ mutually authenticate through *NANG*_*i*_. Table 4 presents the authentication phase of the proposed scheme. The steps involved in this phase are as follows:

- Smart meter *SM*_*i*_ generates a random number w and computes

*C*_*sm*_ = *w.P*_*pub*_ ⊕ *h*_0_(*V*_*m*_ ||*MID*_*i*_)

*CID*_*i*_ = *h*_1_(*w.P*_*pub*_|| *T*_1_) ⊕ *b*_*i*_

*C*_1_ = *h*_1_(*CID*_*i*_ ||*b*_*i*_ ||*w.P*_*pub*_)

- Further *SM*_*i*_ send login request message *M*_1_ = {*C*_*sm*_, *CID*_*i*_, *C*_1_, *T*_1_} to NAN gateway.
- *NANG*_*i*_ receives the message *M*_1_ sent by *SM*_*i*_ and checks the validity. To verify the freshness of the received message, *NANG*_*i*_ takes its current timestamp *T*_2_ and checks that whether *T*_2_ − *T*_1_ ≤ *δ T* or not. Also, *NANG*_*i*_ confirms that between the time (*T*_1_ + *δ T*) and (*T*_1_ − *δ T*) there were no other requests which contains same parameters of *M*_1_ has not received. If these conditions are not true, then the system rejects the request message and drops the session.
- *NANG*_*i*_ computes the following after receiving the login request message:

*w.P*_*pub*_ = *C*_*sm*_ ⊕ *H*_*m*_

*bi* = *h*_1_(*w.P*_*pub*_ ||*T*_1_) ⊕ *CID*_*i*_

*C*_*nan*_ = *C*_*sm*_ ⊕ *H*_*m*_ ⊕ *h*_0_(*V*_*n*_ ||*NID*_*i*_)

*RID*_*s*_ = *h*_1_(*C*_*nan*_ *h*_0_(*V*_*n*_ ||*NID*_*i*_)) ⊕ *b*_*j*_

*C*_2_ = *h*_1_(*C*_1_ ||*T*_2_ ||*C*_*nan*_ *b*_*j*_)

- *NANG*_*i*_ Sends {*CID*_*i*_, *C*_2_, *C*_*nan*_, *RID*_*s*_, *T*_2_, *T*_1_} to the server.
- *SG*_*i*_ receives the request message sent by NAN gateway and checks its validity to make sure that the request is made recently. *SG*_*i*_ takes its present timestamp *T*_3_ and checks whether *T*_3_ − *T*_2_ ≤ *δ T*. If the condition does not satisfy, then *SG*_*i*_ rejects the received message and drops the session. If not, *SG*_*i*_ begins the authentication process.
- Once the request message accepted by the sever, it starts authenticating it. To do that *SG*_*i*_ computes the following: *w.P*_*pub*_ = *C*_*nan*_ ⊕ *H*_*n*_

*b*_*i*_ = *h*_1_(*w.P*_*pub*_|| *T*_1_) ⊕ *CID*_*i*_

*b*_*j*_ = *h*_1_(*C*_*n*_ ||*H*_*n*_) ⊕ *RID*_*s*_

*C*_1_ = *h*_1_(*CID*_*i*_ ||*b*_*i*_ ||*w.P*_*pub*_)

*C*_2_ = *h*_1_(*C*1 ||*T*_2_ ||*C*_*nan*_ ||*b*_*j*_)

- Server compares *C*_2_ with received *C*_2_. If both are equal then the server completes the authentication of *SM*_*i*_ and begins the mutual authentication.
- To perform mutual authentication, server first generates the random number y and computes *C*_*s*_ = *y.P*_*pub*_ ⊕ *h*_2_(*H*_*n*_)

*SK*_*s*_ = *h*_2_(*y.P*_*pub*_ ||*w.P*_*pub*_ ||*b*_*i*_ ||*b*_*j*_ )

*C*_3_ = *h*_2_(*SK*_*s*_ ||*t*_3_ ||*y.P*_*pub*_)

- *SG*_*i*_ sends a mutual authentication message *M*_2_ = {*C*_3_, *C*_*s*_, *T*_3_} to NAN gateway. *NANG*_*i*_ receives *M*_2_ and computes *y.P*_*pub*_ = *C*_*s*_ ⊕ *h*_2_(*h*_0_(*V*_*n*_ ||*NID*_*i*_))

*RID*_*m*_ = *h*_2_(*C*_*m*_|| *H*_*m*_ ) ⊕ *b*_*j*_

*C*_*m*_ = *C*_*s*_ ⊕ *H*_*m*_ ⊕ *h*_0_(*V*_*n*_ ||*NID*_*i*_)

*SK*_*N*_ = *h*_2_(*y.P*_*pub*_|| *w.P*_*pub*_ ||*b*_*i*_ ||*b*_*j*_ )

*C*_4_ = *h*_2_(*C*_3_ ||*T*_4_ ||*C*_*m*_ ||*b*_*j*_)

Sends {*C*_*s*_, *T*_3_, *C*_4_, *C*_*m*_, *T*_4_, *RID*_*m*_} to the *SM*_*i*_

- Smart meter receives mutual authentication message from NAN gateway and computes

*y.P*_*pub*_ = *C*_*s*_ ⊕ *h*_0_(*V*_*m*_ ||*MID*_*i*_)

*b*_*j*_ = *h*_1_(*C*_*m*_ *h*_0_(*V*_*m*_ ||*MID*_*i*_)) ⊕ *RID*_*m*_

*SK*_*M*_ = *h*_2_(*y.P*_*pub*_ ||*w.P*_*pub*_ ||*b*_*i*_ ||*b*_*j*_ )

*C*_3_ = *h*_2_(*SK*_*M*_ ||*T*3 ||*y.P*_*pub*_ )

*C*4 = *h*_2_(*C*_3_ ||*T*_4_ ||*C*_*m*_ ||*b*_*j*_)

- *SM*_*i*_ Compares *C*_4_ with received *C*_4_ if both are equal smart meter, NAN gateway, and the server establishes the connection successfully.
- Further communications will be done through the shared session keys. The session keys are For smart meter, *SK*_*M*_ = *h*_2_(*y.P*_*pub*_ ||*w.P*_*pub*_ ||*b*_*i*_ ||*b*_*j*_)

For NAN gateway *SK*_*N*_ = *h*_2_(*y.P*_*pub*_ ||*w.P*_*pub*_ ||*b*_*i*_ ||*b*_*j*_ )

For Server *SK*_*s*_ = *h*_2_(*y.P*_*pub*_ ||*w.P*_*pub*_ ||*b*_*i*_ ||*b*_*j*_ ).

**Table 4:** Login and authentication phase of the proposed scheme.

Smart meter	NAN gateway	SCADA control center/server
Generates w and computes		
*C*_*sm*_ = *w.P*_*pub*_ ⊕ *h*_0_(*V*_*m*_ ||*MID*_*i*_)		
*CID*_*i*_ = *h*_1_(*w.P*_*pub*_|| *T*_1_) ⊕ *b*_*i*_		
*C*_1_ = *h*_1_(*CID*_*i*_ ||*b*_*i*_||*w.P*_*pub*_)		
Sends *M*_1_ = {*C*_*sm*_, *CID*_*i*_, *C*_1_, *T*_1_} to NAN gateway.		
{*M_1_ = C_sm_,CID_i_,C_1_,T_1_*} −−−−−−−−−−−−−−→		
	Receives the message sent and checks *T*_2_ − *T*_1_ ≤ *δ T*.	
	Computes	
	*w.P*_*pub*_ = *C*_*sm*_ ⊕ *H*_*m*_	
	*b*_*i*_ = *h*_1_(*w.P*_*pub*_|| *T*_1_) ⊕ *CID*_*i*_	
	*C*_*nan*_ = *C*_*sm*_ ⊕ *H*_*m*_ ⊕ *h*_0_(*V*_*n*_ ||*NID*_*i*_)	
	*RID*_*s*_ = *h*_1_(*C*_*nan*_ ||*h*_0_(*V*_*n*_ ||*NID*_*i*_)) ⊕ *b*_*j*_	
	*C*_2_ = *h*_1_(*C*_1_|| *T*_2_|| *C*_*nan*_ ||*b*_*j*_)	
	Sends {*CID*_*i*_, *C*_2_, *C*_*nan*_, *RID*_*s*_, *T*_2_, *T*_1_} to the *SG*_*i*_.	
	*CID_i_,C_2_,C_nan_,RID_s_,T_2_,T_1_* −−−−−−−−−−−−−−−→	
		Receives the request message and checks whether *T*_3_ − *T*_2_ ≤ *δ T*.
		Computes the following:
		*w.P*_*pub*_ = *C*_*nan*_ ⊕ *H*_*n*_
		*b*_*i*_ = *h*_1_(*w.P*_*pub*_|| *T*_1_) ⊕ *CID*_*i*_
		*b*_*j*_ = *h*_1_(*C*_*n*_|| *H*_*n*_) ⊕ *RID*_*s*_
		*C*_1_ = *h*_1_(*CID*_*i*_|| *b*_*i*_ ||*w.P*_*pub*_ )
		*C*_2_ = *h*_1_(*C*1|| *T*_2_ ||*C*_*nan*_ ||*b*_*j*_ )
		Checks *C*_2_ ? = *C*_2_ If both are equal then.
		Generates y and computes
		*C*_*s*_ = *y.P*_*pub*_ ⊕ *h*_2_(*H*_*n*_)
		*SK*_*s*_ = *h*_2_(*y.P*_*pub*_|| *w.P*_*pub*_ ||*b*_*i*_ ||*b*_*j*_ )
		*C*_3_ = *h*_2_(*SK*_*s*_|| *t*_3_ ||*y.P*_*pub*_)
		Sends *M*_2_ = {*C*_3_, *C*_*s*_, *T*_3_}.
		{*M_2_ = C_3_,C_s_,T_3_*} ←−−−−−−−−−−
	Receives *M*_2_ and computes	
	*y.P*_*pub*_ = *C*_*s*_ ⊕ *h*_2_(*h*_0_(*Vn* ||*NID*_*i*_))	
	*RID*_*m*_ = *h*_2_(*C*_*m*_|| *H*_*m*_ ) ⊕ *b*_*j*_	
	*C*_*m*_ = *C*_*s*_ ⊕ *H*_*m*_ ⊕ *h*_2_(*h*_0_(*Vn*|| *NID*_*i*_))	
	*SK*_*N*_ = *h*_2_(*y.P*_*pub*_ ||*w.P*_*pub*_ ||*b*_*i*_ ||*b*_*j*_ )	
	*C*_4_ = *h*_2_(*C*_3_ ||*T*_4_ ||*C*_*m*_ ||*b*_*j*_)	
	Sends {*C*_*s*_, *T*_3_, *C*_4_, *C*_*m*_, *T*_4_, *RID*_*m*_} to the *SM*_*i*_	
	{*C_s_,T_3_,C_4_,C_m_,T_4_,RID_m_*} ←−−−−−−−−−−−−−−	
Receives mutual authentication message and computes		
*y.P*_*pub*_ = *C*_*s*_ ⊕ *h*_0_(*V*_*m*_|| *MID*_*i*_)		
*b*_*j*_ = *h*_1_(*C*_*m*_|| *h*_0_(*V*_*m*_|| *MID*_*i*_)) ⊕ *RID*_*m*_		
*SK*_*M*_ = *h*_2_(*y.P*_*pub*_ ||*w.P*_*pub*_ ||*b*_*i*_ ||*b*_*j*_ )		
*C*_3_ = *h*_2_(*SK*_*M*_ ||*T*3 ||*y.P*_*pub*_ )		
*C*4 = *h*_2_(*C*_3_ ||*T*_4_ ||*C*_*m*_ ||*b*_*j*_)		
Smart meter session key	NAN Gateway session key	Server session key
*SK*_*M*_ = *h*_2_(*y.P*_*pub*_ ||*w.P*_*pub*_ ||*b*_*i*_ ||*b*_*j*_ )	*SK*_*N*_ = *h*_2_(*y.P*_*pub*_ ||*w.P*_*pub*_ ||*b*_*i*_ ||*b*_*j*_ )	*SK*_*s*_ = *h*_2_(*y.P*_*pub*_ ||*w.P*_*pub*_ ||*b*_*i*_ ||*b*_*j*_ )

## Cryptanalysis of the proposed scheme

This section presents the cryptanalysis of the proposed multistage authentication scheme. This analysis helped us to prove the sensitivity of the obtained results where stored and communication parameters does not impact on the systems privacy and the security.

### Resiliency against replay attack

Resiliency against replay attacks can be possible only when the server verifies the freshness of the login message before beginning the authentication. In the proposed scheme, a timestamp has been used to check the validity of the login request message. Suppose a participant receives the message contains timestamp T, then the participant takes its current timestamp T and checks the condition T′ - T ≤ *δ*T. If this condition is satisfied, the next step would be to proceed else participant drops the session.

Let us assume that adversary steals the previous successfully authenticated login request message {*C*_*sm*_, *CID*_*i*_, *C*_1_, *T*_1_} sent from *SM*_*i*_ to *NANG*_*i*_ and {*CID*_*i*_, *C*_2_, *C*_*nan*_, *RID*_*s*_, *T*_2_, *T*_1_} sent from *NANG*_*i*_ to *SG*_*i*_. Attacker trying perform replay attack by resending stolen login request message. Here, both *NANG*_*i*_ and *SG*_*i*_ first verifies the message freshness by checking the validity of the time stamp. To verify the freshness, *NANG*_*i*_ takes its current timestamp *T*_2_ and checks whether *T*_2_ − *T*_1_ ≤ *δ T* or not. Also, *NANG*_*i*_ confirms that there were no other requests with parameters have been received between the time (*T*_1_ + *δ T*) and (*T*_1_ − *δ T*). If the timestamp *T*_1_ is not modified in the login request message, the condition *T*_2_ − *T*_1_ ≤ *δ T* will definitely fails and *NANG*_*i*_ drops the session. This procedure also happens when *SG*_*i*_ receives login request message from *NANG*_*i*_. Therefore, the proposed scheme resists the replay attack.

### Resiliency against insider attack

The proposed distributed multistage authentication scheme does not store all the information in a single system/server. The registration of *SM*_*i*_ is with *NANG*_*i*_ and the registration of *NANG*_*i*_ will be done by *SG*_*i*_. This process distributes the necessary credentials to *SM*_*i*_, *NANG*_*i*_ and *SG*_*i*_. To perform any attack, insider must know parameters stored in other two participants *SM*_*i*_ and *NANG*_*i*_. Hence the proposed scheme ensures resiliency against insider attack.

### Resiliency against impersonation attack

When the communication has taken place in an open channel, the attacker can intercept the sent/received messages and perform an impersonation attack. Assume that an attacker ${\tf="script" \char "41}$ intercepts the login and authentication messages {*C*_*sm*_, *CID*_*i*_, *C*_1_, *T*_1_}, {*CID*_*i*_, *C*_2_, *C*_*nan*_, *RID*_*s*_, *T*_2_, *T*_1_}, {*C*_3_, *C*_*s*_, *T*_3_}, and {*C*_*s*_, *T*_3_, *C*_4_, *C*_*m*_, *T*_4_, *RID*_*m*_} to perform impersonation attack. In the proposed scheme, computation of the communication parameters are depending upon the random nonces *w*, *y* and the parameters {*NID*_*i*_, *b*_*j*_, *V*_*n*_} stored in *SM*_*i*_. Since the random nonce and stored parameters do not communicate in the open channel as plain text, attacker ${\tf="script" \char "41}$ cannot get the knowledge of it. Therefore ${\tf="script" \char "41}$ cannot modify the login request parameters to impersonate the user.

### Resiliency against man-in-the-middle attack

A man-in-the-middle attack can be possible when the adversary successfully authenticates with the server or calculate the session key correctly by intercepting communication messages. In the proposed scheme, to mitigate this attack we have applied two types of mechanism. First and foremost the communication message parameters of the proposed scheme are computed depending upon the generated random nonces *w*, *y*. Usage of random nonce prevents the impersonation and preserves the secrecy of the session key. Secondly, a timestamp has been used to check the validity of the login request message in every session. As said in “Resiliency Against Replay Attack”, checking the message freshness resists the replay attack. Hence, the attacker cannot authenticate himself by intercepting the communication messages.

### Provides perfect forward secrecy

In the proposed scheme, session key calculated by *SK* = *h*_2_(*y.P*_*pub*_ ||*w.P*_*pub*_ ||*b*_*i*_ ||*b*_*j*_). An adversary ${\tf="script" \char "41}$ cannot compute *SK* by intercepting the mutual authentication messages {*C*_3_, *C*_*s*_, *T*_3_}, and {*C*_*s*_, *T*_3_, *C*_4_, *C*_*m*_, *T*_4_, *RID*_*m*_}. An attacker must have the knowledge of random nonces *w*, *y*, *b*_*i*_ and *b*_*j*_ to calculate *SK*. The proposed scheme cannot share random nonce as plain text over the public channel. Therefore to calculate *SK*, an adversary has to guess all random nonces at the same time, which is not possible. Hence the proposed scheme provides perfect forward secrecy.

### Provides device anonymity

In the proposed scheme, device anonymity is preserved while login into the system. A dynamic identity *CID*_*i*_ = *h*_1_(*w.P*_*pub*_ ||*T*_1_) ⊕ *b*_*i*_ has been calculated every session. The computation of the *CID*_*i*_ is depending on the random nonce *w*. Therefore the dynamic identity is different at every login attempt. Hence the proposed scheme provides reveal the device identity *MID*_*i*_.

### Provides data confidentiality

In the proposed scheme, the confidentiality of the communicated messages has been preserved even after authentication. The secrecy of the message after authentication can be achieved by the session key. In the proposed scheme, if the attacker tries to steal any message communicated between *SM*_*i*_, *NANG*_*i*_ and *SG*_*i*_ cannot decrypt without *SK*. As explained in “Provides Perfect Forward Secrecy”, the session key cannot be compromised with the adversary. Moreover, during authentication, after successful verification of the login request *SM*_*i*_, *NANG*_*i*_ and *SG*_*i*_ will mutually authenticate each other and compute the session key. This creates a secure channel over an insecure environment. So, the communicated message remains confidential between *SM*_*i*_, *NANG*_*i*_ and *SG*_*i*_.

From the cryptanalysis result of the proposed scheme it can be proved that the data confidentiality, sensitivity and the security has been achieved by resisting the possible attacks on the network.

## Formal security proof

This section presents the formal security analysis to prove that the proposed scheme is secure against the adversary modeled in Odelu et al. (2016) and Tsai & Lo (2015) proposed by Canetti & Krawczyk (2001). According to this mode, adversary ${\tf="script" \char "41}$ has full control over the transmission channel. Therefore ${\tf="script" \char "41}$ can eavesdrop, intercept, alter the communication messages, and knows all the public parameters. The adversary cannot access the secret parameter directly but can construct queries to capture the information leakage.

### Participants

A participant in the entity takes part in the authentication process. In the proposed scheme, there are three participants are involved in performing the authentication, which is named as *SM*_*i*_, *NANG*_*i*_, and *SG*_*i*_, where *SM*_*i*_ is a smart meter, *NANG*_*i*_ for the Neighborhood Area Network gateway, and *SG*_*i*_ is the SCADA control center/Server. Each participant have multiple instances to run the scheme parallelly. The instances are represented as *SM*^*i*^, *NANG*^*i*^, and *SG*^*i*^, where ‘i’ represents the *i*^*t*^*h* instance of the participants (Tsai & Lo, 2015).

### Queries

In CK—model, adversary ${\tf="script" \char "41}$ can construct the queries to perform the attacks. The possible queries made by ${\tf="script" \char "41}$, and the attacks committed with the constructed query are illustrated below:

- *Execute*(*SM*^*i*^, *NANG*^*i*^, *SG*^*i*^): This oracle query construct the passive attacks by eavesdropping the successful execution done between the participants *SM*_*i*_, *NANG*_*i*_, *SG*_*i*_. Here, ${\tf="script" \char "41}$ simulates the login and authentication phase and gets the messages communicated between the participants.
- *Send*(*SM*^*i*^/*NANG*^*i*^/*SG*^*i*^, *M*): ${\tf="script" \char "41}$ formulatess this query to perform active attacks. Adversary sends message to *SM*^*i*^/*NANG*^*i*^/*SG*^*i*^ and receives the response from *SM*^*i*^/*NANG*^*i*^/*SG*^*i*^.Also, ${\tf="script" \char "41}$ can intercept communication channel and modify the communicated messages and gets the reply in return.
- *EKeyReveal* (*SM*^*i*^/*SG*^*i*^): This query allows adversary to obtain the session state ephemeral secret key information held by the instance *SM*^*i*^/*NANG*^*i*^/*SG*^*i*^.
- *SKReveal* (*SM*^*i*^/*SG*^*i*^): This query allows adversary to get the session key held by the instance *SM*^*i*^/*NANG*^*i*^/*SG*^*i*^.
- *Corrupt* (*SM*^*i*^/*SG*^*i*^): This query express the notion of perfect forward secrecy where long term secret key can be compromise with ${\tf="script" \char "41}$ to get the session key on the oracle *SM*^*i*^/*NANG*^*i*^/*SG*^*i*^.
- *Test*(*SM*^*i*^/*SG*^*i*^): This single query can be constructed by adversary at most once. It models the semantic security of the session. Here, ${\tf="script" \char "41}$ returns the session key of *SM*^*i*^/*NANG*^*i*^/*SG*^*i*^ or a random string with an equal bit length of the session key. This result is depending upon tossing a coin *b*. If *b* = 1, the adversary gets the original session key. Else ${\tf="script" \char "41}$ gets a random string with the same length as the real session key.

Before presenting the security proof, it is necessary to describe some definitions, which are given below:

- Partnering: Two entities are called to be partners when they are accepted and shared a commen session key. In the proposed scheme, *SM*_*i*_, *NANG*_*i*_ and *SG*_*i*_ are partners only if *MID*_*i*_ = *NID*_*j*_ = *SID*_*k*_, and *SK*_*SMi*_ = *SK*_*NANGi*_ = *SK*_*SGi*_.
- Freshness: It is related to the session key. Here, oracle constructs the session key. We can say that the constructed session key is fresh if the instance meets the following conditions.

1. When there is no *Reveal* query is done by *SM*_*i*_, *NANG*_*i*_ and *SG*_*i*_, sission key *SK*_*i*_ should not be null.
2. *Send*(*SM*_*i*_, *M*), *Send*(*NANG*_*i*_, *M*) or *Send*(*SG*_*i*_, *M*) should be asked after modelling the *Currupt* query

- Semantic Security: The goal of semantic security is to guess the bit ′*b*′, which is involved in the *Test*(*SM*^*i*^/*SG*^*i*^) query. Consider an event *S*() that the adversary ${\tf="script" \char "41}$ guess the bit *b* correctly. Let *SM*_*i*_, *NANG*_*i*_ and *SG*_*i*_ oracles are considered as partners when authenticating each other and share a common session key. The adversary’s goal is to differentiate the session key from a random key. ${\tf="script" \char "41}$ can model many Test queries for *SM*^*i*^ or *SG*^*i*^. Consider queries, for instance, *SM*^*i*^. Further, *SM*^*i*^ toss a coin b. If b=1, the adversary gets the original session key. Else A gets a random string with the same length as the real session key.

Let *Pr*[*S*] denotes the game-winning probability of ${\tf="script" \char "41}$. The advantage of the Adversary A against breaking the semantic security of the proposed scheme is *Adv*^*AKE*^_*P*_ (*A*) = |2*Pr*[*Succ*] − 1|.

### Computational problem

It is essential to describe the details of the computational problem where the security proof relies upon.

- Elliptic curve computational Diffie-Hellman problem (ECDH): Let *P*, *xP*, *yP* ∈ *E*_*p*_ where $a,b \in Z_q^*$, then it is hard to compute *xyP* in polynomial time without knowledge of *x* or *y*.
- Elliptic curve discrete logarithm problem (ECDLP): It says that when *G* ∈ *E*_*p*_(*x*, *y*) of order *n* and *G* = *kP* ∈ *E*_*p*_(*x*, *y*), it is computationally infeasible to compute *k* in polynomial-time.
- Reversing One way Hash function: Let *H*(.) is a one way hash function, then it is computationally hard to get *x* from *H*(*x*). Also it is hard to find *x* where *H*(*x*) = *H*(*x*).

### Security proof

**Theorem 1** *Let E*_*p*_ *over the finite field F*_*p*_ *with a large prime number p and* ${\tf="script" \char "44}$ *be the finite set of password. Consider* ${\tf="script" \char "41}$ *is a adversary running in a polynomial time to perfom security attack on the proposed scheme. Let Adv*^*AKE*^_*SC*_ *is the advantage of the* ${\tf="script" \char "41}$ *against the proposed scheme and advantage of* ${\tf="script" \char "41}$ *that solves ECDH in E*_*p*_. *While performing the attackes over the proposed scheme* ${\tf="script" \char "41}$ *makes q*_*send*_ *Send queries, q*_*hsh*_ *hash oracles, and q*_*exe*_ *Execute queries within the time t. The advantage of* ${\tf="script" \char "41}$ *will be*

$$Adv_{AKE}^{SG} \le \displaystyle{{{{({q_{send}} + {q_{exe}})}^2}} \over {2n}} + \displaystyle{{{{({q_{hsh}})}^2}} \over {{2^k}}} + \displaystyle{{{q_{send}}} \over {{2^k} + n}} + {q_{hsh}}.Adv_{EC}^{ECDH}(t + ({q_{exe}} + {q_{send}}){T_{EC}})$$

*Proof:* The queries constructed by ${\tf="script" \char "41}$ has been presented in Tables 5 and 6. Based on the queries build by ${\tf="script" \char "41}$, the proof is presented. The sequence of experiments from *Experiment*_0_ to *Experiment*_4_ defines the proof of the proposed authenticaion scheme. Let *Succ*_*n*_ denotes the event that occures after the *Test* query made by adversary while guessing the bit *b* correctly.

**Table 5:** Simulation of send query.

For a hash oracle *h*(*i*, *q*) where *i* = 0, 1, 2 if (*i*, *q*, *h*) ∈ *L*_*h*_ Return *h*
Else, Choose *h* and add to *L*_*h*_ as (*i*, *q*, *h*)
For *Execute* (*SM*^*i*^, *NANG*^*i*^, *SG*^*i*^) query
$(CI{D_i},{C_2},{C_{nan}},RI{D_s},{T_2},{T_1}) \leftarrow Send({C_{sm}},CI{D_i},{C_1},{T_1})$
$({C_3},{C_s},{T_3}) \leftarrow Send(CI{D_i},{C_2},{C_{nan}},RI{D_s},{T_2},{T_1})$
$({C_s},{T_3},{C_4},{C_m},{T_4},RI{D_m}) \leftarrow Send({C_3},{C_s},{T_3})$
*Return* (*C*_*sm*_, *CID*_*i*_, *C*_1_, *T*_1_), (*CID*_*i*_, *C*_2_, *C*_*nan*_, *RID*_*s*_, *T*_2_, *T*_1_), (*C*_3_, *C*_*s*_, *T*_3_), (*C*_*s*_, *T*_3_, *C*_4_, *C*_*m*_, *T*_4_, *RID*_*m*_)
For *EKeyReveal* (*SM*^*i*^/*SG*^*i*^) query return Ephermal Secret key *w* from *SM*^*i*^ and *y* from *SG*^*i*^
For *SKReveal* (*SM*^*i*^/*SG*^*i*^) query return static private key *s* from *SM*^*i*^ or *SG*^*i*^
For *Test*(*SM*^*i*^/*NANG*^*i*^/*SG*^*i*^) query
$S{K_p} \leftarrow Revel(S{M^i}/NAN{G^i}/S{G^i})$
$b \leftarrow \{ 0,1\}$
$S{K_p} \leftarrow {\{ 0,1\} ^{k^\prime}}$
For *Corrupt*() query
If *P* = *P*_*i*_
Return *RPW*_*i*_ or *A*_*i*_
Else if *P* = *S*
Return *A*_*i*_

**Table 6:** Simulation of execute, revel and test query.

For *Send* (*P*^*i*^, *Start*) query
Generate random number *w* and computes *C*_*sm*_ = *w.P*_*pub*_ ⊕ *h*_0_(*V*_*m*_|| *MID*_*i*_), *CID*_*i*_ = *h*_1_(*w.P*_*pub*_ ||*T*_1_) ⊕ *b*_*i*_,
*C*_1_ = *h*_1_(*CID*_*i*_ ||*b*_*i*_ ||*w.P*_*pub*_), Return (*C*_*sm*_, *CID*_*i*_, *C*_1_, *T*_1_).
For *Send* (*C*_*sm*_, *CID*_*i*_, *C*_1_, *T*_1_) query
*w.P*_*pub*_ = *C*_*sm*_ ⊕ *H*_*m*_, *bi’* = *h*_1_(*w.P*_*pub*_ ||*T*_1_) ⊕ *CID*_*i*_ *C*_*nan*_ = *C*_*sm*_ ⊕ *H*_*m*_ ⊕ *h*_0_(*V*_*n*_|| *NID*_*i*_)
*RID*_*s*_ = *h*_1_(*C*_*nan*_ ||*h*_0_(*V*_*n*_ ||*NID*_*i*_)) ⊕ *b*_*j*_ *C*_2_ = *h*_1_(*C*_1_ ||*T*_2_ ||*C*_*nan*_ ||*b*_*j*_) Return (*CID*_*i*_, *C*_2_, *C*_*nan*_, *RID*_*s*_, *T*_2_, *T*_1_)
For *Send* (*CID*_*i*_, *C*_2_, *C*_*nan*_, *RID*_*s*_, *T*_2_, *T*_1_) query
*w.P*_*pub’*_ = *C*_*nan*_ ⊕ *H*_*n*_ *b*_*i’*_ = *h*_1_(*w.P*_*pub*_ ||*T*_1_) ⊕ *CID*_*i*_ *b*_*j*_ = *h*_1_(*C*_*n*_ ||*H*_*n*_) ⊕ *RID*_*s*_
*C*_1’_ = *h*_1_(*CID*_*i*_ ||*b*_*i’*_ ||*w.P*_*pub*_) *C*_2’_ = *h*_1_(*C*1’||*T*_2_ ||*C*_*nan*_ ||*b*_*j*_)
If *C*_2’_ = *C*_2_
Generate random number *y* and computes
*C*_*s*_ = *y.P*_*pub*_ ⊕ *h*_2_(*H*_*n*_) *SK*_*s*_ = *h*_2_(*y.P*_*pub*_ ||*w.P*_*pub’*_ ||*b*_*i*_‘||*b*_*j’*_) *C*_3_ = *h*_2_(*SK*_*s*_ *t*_3_ *y.P*_*pub*_)
Return (*C*_3_, *C*_*s*_, *T*_3_)
For *Send* (*C*_3_, *C*_*s*_, *T*_3_) query
*y.P*_*pub*_ = *C*_*s*_ ⊕ *h*_2_(*h*_0_(*Vn* ||*NID*_*i*_)) *RID*_*m*_ = *h*_2_(*C*_*m*_ ||*H*_*m*_ ) ⊕ *b*_*j*_ *C*_*m*_ = *C*_*s*_ ⊕ *H*_*m*_ ⊕ *h*_2_(*h*_0_(*Vn* ||*NID*_*i*_))
*SK*_*N*_ = *h*_2_(*y.P*_*pub*_ ||*w.P*_*pub’*_ ||*b*_*i’*_|| *b*_*j’*_ ) *C*_4_ = *h*_2_(*C*_3_ ||*T*_4_ ||*C*_*m*_ ||*b*_*j*_)
Return (*C*_*s*_, *T*_3_, *C*_4_, *C*_*m*_, *T*_4_, *RID*_*m*_)
For *Send* (*C*_3_, *C*_*s*_, *T*_3_) query
*y.P*_*pub’*_ = *C*_*s*_ ⊕ *h*_0_(*V*_*m*_ ||*MID*_*i*_) *b*_*j’*_ = *h*_1_(*C*_*m*_ ||*h*_0_(*V*_*m*_ ||*MID*_*i*_)) ⊕ *RID*_*m*_ *SK*_*M*_ = *h*_2_(*y.P*_*pub’*_|| *w.P*_*pub*_ ||*b*_*i*_ ||*b*_*j’*_ )
*C*_3’_ = *h*_2_(*SK*_*M*_ ||*T*3 ||*y.P*_*pub*_’) *C*4’ = *h*_2_(*C*_3’_|| *T*_4_ ||*C*_*m*_ ||*b*_*j*_)
If *C*4 = *C*4 *SM*_*i*_, *NANG*_*i*_ and *SG*_*i*_ Else Terminated

*Experiment 0*: This experiment corresponds to the real experiment in the random oracle model. By the definition, we have

$$Adv_{AKE}^{SG} \le 2Pr[Suc{c_0}] - 1$$

*Experiment* 1: This experiment simulates *H*_0_, *H*_1_, *H*_2_ by maintaining two hash list *L*_*h*_ and *L*_*h*_. Here, *L*_*h*_ stores the oracle for *H*_0_, *H*_1_, *H*_2_ and *L*_*h*_ is for queries asked by ${\tf="script" \char "41}$. The simulation queries are presented in Table 5. From the simulation, it is observed that the transcript distribution of *Experiment* 0 and *Experiment* 1 are indistinguishable. Hence we have

$$Pr[Suc{c_0}] = Pr[Suc{c_1}]$$

*Experiment* 2: This experiment simulates the oracle of *Experiment* 1 except the collisions occurs in the transcripts and hash queries by the adversary. In other words, the experiment aims to avoid the collision occurring in *C*_1_, *C*_3_, *C*_*sm*_ and *C*_*s*_. The *Experiment* 1 and *Experiment* 2 are indistinguishable until the collision takes place. Since, *w* and *y* are randomly chosen, according to the birthday paradox, probability of the collision occurrence is at most (*q*_*send*_ + *q*_*exe*_)^2^/2*n*. Also, the probability of the occurrence of the collision in the output of the hash oracle is at most (*q*_*hsh*_)^2^/2^*k*^. Hence we have

(1) $$|Pr[Suc{c_2}] - Pr[Suc{c_1}]| \le \displaystyle{{{{({q_{send}} + {q_{exe}})}^2}} \over {2n}} + \displaystyle{{{{({q_{hsh}})}^2}} \over {{2^k}}}$$

*Experiment 3*: This experiment aborts the scheme if the adversary succeeds in guessing the authentication value *C*_1_ and *C*_3_ without making the hash query. Since, *Experiment* 3 and *Experiment* 2 are indistinguishable unless smart grid *SG*_*i*_ rejects *C*_1_ or smart meter *SM*_*i*_ rejects the authentic value *C*_3_. Hence we have

(2) $$|Pr[Suc{c_3}] - Pr[Suc{c_2}]| \le \displaystyle{{{q_{send}}} \over {{2^k} + n}}$$

*Experiment 4*: This experiment considers the session key security. ${\tf="script" \char "41}$ cannot obtain the previous session key when ${\tf="script" \char "41}$ has {*w*, *y*, *s*, *P*} but not (*w*, *s*, *P*) and (*y*, *s*, *P*). The aim of ${\tf="script" \char "41}$ is to compute the session key *SK*_*i*_ = *h*_2_(*y.s.P w.s.P b*_*i*_ *b*_*j*_) by asking *Execute*(*SM*^*i*^, *NANG*^*i*^, *SG*^*i*^) queries and corresponding hash queries in the four cases.

case 1: ${\tf="script" \char "41}$ queries *Corrupt* (*SM*^*i*^) and *Corrupt* (*SG*^*i*^) to get static private key *s* to compute the session key *SK*_*i*_. To derive the session key ${\tf="script" \char "41}$ should get *w* and *y*.

case 2: ${\tf="script" \char "41}$ queries *EKeyReveal* (*SM*^*i*^) and *EKeyReveal* (*SG*^*i*^) to get ephermal private key *w* and *y*. But ${\tf="script" \char "41}$ will not get the static key *s*.

case 3: ${\tf="script" \char "41}$ queries *EKeyReveal* (*SM*^*i*^) and *Corrupt* (*SG*^*i*^) and returns *w* and *s*. But ${\tf="script" \char "41}$ will not get *y*.

case 4: ${\tf="script" \char "41}$ queries *Corrupt* (*SM*^*i*^) and *EKeyReveal* (*SG*^*i*^) and returns *y* and *s*. But ${\tf="script" \char "41}$ will not get *x*.

In all the above four cases, ${\tf="script" \char "41}$ will get insufficient information to compute *SK*_*i*_ without solving the ECDH. The *Experiment* 3 and *Experiment* 4 are indistinguishable until the ECDH assumption is true. Hence we have

(3) $$|Pr[Suc{c_4}] - Pr[Suc{c_3}]| = {q_{hsh}}.Adv_{EC}^{ECDH}(t + ({q_{exe}} + {q_{send}}){T_{EC}})$$

Now, ${\tf="script" \char "41}$ has to guess *b* to achieve the experiment by the *Test* query. It is clear that *Pr*[*Succ*_4_] = 1/2.

## Result of formal security verification using avispa tool

The results of the security verification using the AVISPA tool are presented in this section. AVISPA is a protocol analysis tool that provides a platform to implement the schemes and verify its security. To implement schemes, AVISPA uses High-Level Protocol Specification Language (HLPSL). It is a role-based language where every participant in the network plays a role during the execution. In HLPSL, the Doley-Yao model has been used to build the intruder. During the execution of schemes, the HLPSL code is converted into an Intermediate Format (IF) through a translator called hlpsl2if. Further, the translated IF is read by backends and analyses security goals. There are four backends are used in AVISPA used for security analysis known as On-the-fly Model-Checker (OFMC) (Basin, Mödersheim & Vigano, 2005), CL-AtSe (Constraint Logic-based Attack Searcher) (Turuani, 2006), SAT-based Model checker (Armando & Compagna, 2004) and Tree Automata based on Automatic Approximations for the Analysis of Security Protocols (TA4SP) (Boichut et al., 2004). If the scheme achieves all defined goals, then the output is given as SAFE else, the output will be UNSAFE.

**Figure 2:**
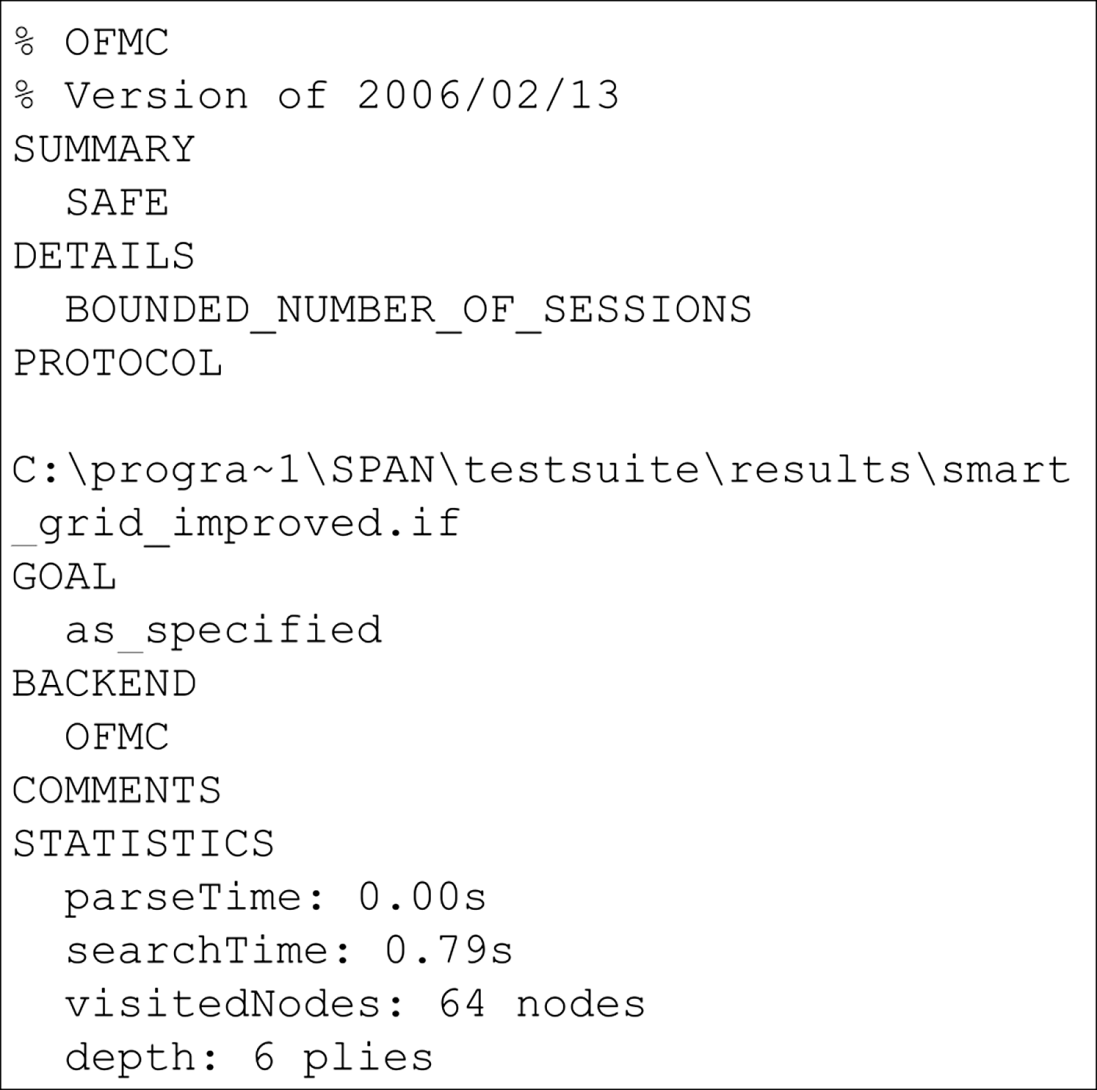
OFMC simulation result of proposed scheme.

**Figure 3:**
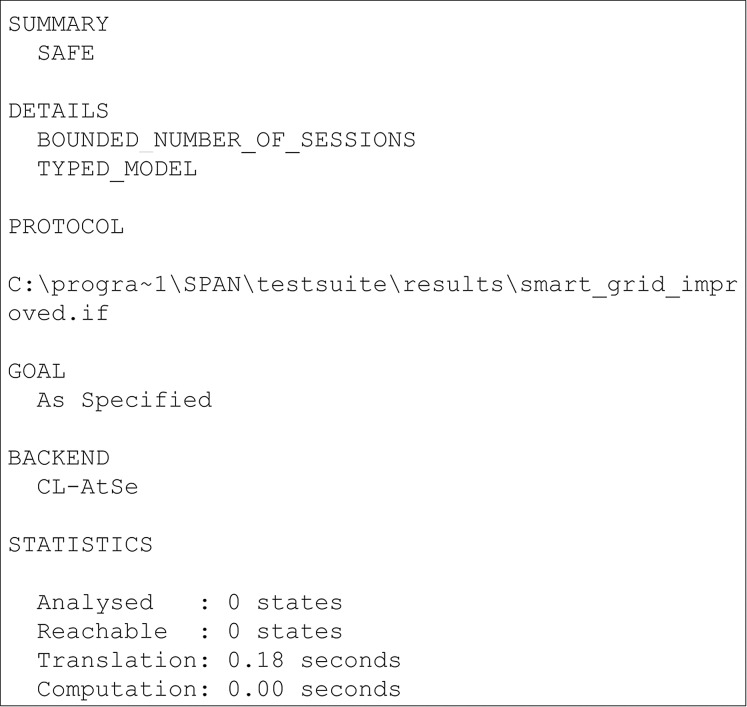
CLAtSe simulation result of proposed scheme.

The results of the security verification obtained from the AVISPA tool are presented in Figs. 2 and 3. The presented results are obtained through OFMC, and CLAtSe back ends. The other two backends SATMC and TA4SP, do not support the XOR feature. Hence the results were received as “Inconclusive”. Therefore the OFMC and CLAtSe results are considered and claimed that the obtained result is SAFE against the intruder. Hence, we can clearly say that the proposed scheme achieves all the specified goals and remains secure against all the attacks.

## Efficiency analysis

This section focuses on the efficiency analysis of the proposed scheme. The analysis mainly focuses on calculating the computation and communication costs and comparing the result with related authentication schemes like Tsai & Lo (2015), Wazid et al. (2017), Odelu et al. (2016), Gope & Sikdar (2018), Kumar et al. (2018), Zhang et al. (2019), Ma et al. (2019), Wu et al. (2019), Li et al. (2019) and Khan, Kumar & Ahmad (2019).

### Computation cost analysis

The proposed scheme’s computation cost has been calculated and compared with the other schemes. We have presented the required computation cost and estimated execution time of the proposed scheme in Smart meter, NAN gateway, and SCADA control center/Server. To measure the execution time, we have taken the results obtained by Gope & Sikdar (2018). According to the results presented by Gope & Sikdar (2018), the required execution time for one computational parameter on Smart meter, NAN gateway, and SCADA control center/Server is given in Table 7. The computational parameters are defined as follows: *T*_*h*_: The time for executing a one-way hash operation, *T*_*s*_: The time for symmetric key encryption/decryption execution operation, *T*_*mp*_: scalar multiplication operation of an elliptic curve, *T*_*a*_: Point addition operation of an elliptic curve, *T*_*b*_: The time to perform one bilinear pairing operation, *T*_*e*_: The time to complete the modular exponential operation, *T*_*fe*_: The time to perform fuzzy extractor Gen(·)/Rep(·) operation, *T*_*PUF*_: The time to perform Operation of PUF circuit.

**Table 7:** Execution time of cryptographic operations.

Operation	*SM*_*i*_ & *NANG*_*i*_	*SG*_*i*_
*T*_*h*_	0.026 ms	0.011 ms
*T*_*s*_	0.079 ms	0.041 ms
*T*_*mp*_	5.9 ms	2.6 ms
*T*_*e*_	7.86 ms	2.34 ms
*T*_*b*_	9.23 ms	3.78 ms
*T*_*PUF*_	0.12 ms	–
*T*_*fe*_	3.28 ms	1.17 ms

**Figure 4:**
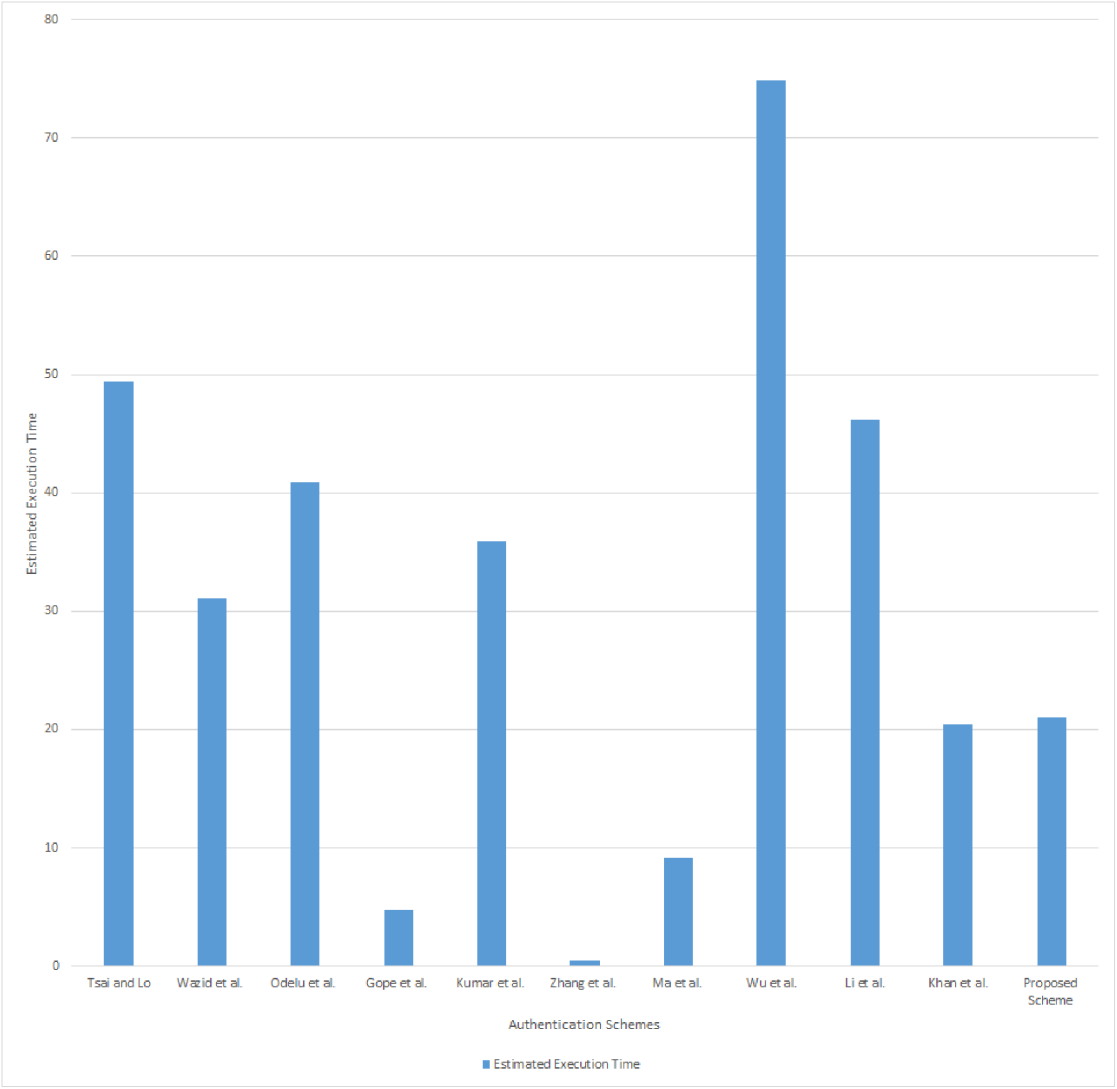
Computation cost analysis.

The computation cost analysis results are given in the Table 8 and the estimated execution time analysis is presented Fig. 4. The computation cost of the proposed scheme is the sum of the costs of *SM*_*i*_, *NANG*_*i*_, and *SG*_*i*_, which is 2*T*_*mp*_ + 12*T*_*h*_, 1*T*_*mp*_ + 12*T*_*h*_, and 1*T*_*mp*_ + 7*T*_*h*_, respectively. The total computation cost of the proposed scheme is 4*T*_*mp*_ + 32*T*_*h*_. Estimated execution time of the proposed scheme in the *SM*_*i*_ and *NANG*_*i*_ is (3 × 5.9 ms) + (24 × 0.026 ms) = 18.324 ms. The estimated execution time in the *SG*_*i*_ side is (1 × 2.6 ms) + (7 × 0.011 ms) = 2.677 ms. The total estimated execution time of the proposed scheme is 21.001 ms.

**Table 8:** Computation cost analysis.

Schemes	*SM*_*i*_	*NANG*_*i*_	*SG*	Total	Expected time
Tsai & Lo (2015)	4*T*_*mp*_ + *T*_*e*_ + *T*_*h*_	–	2*T*_*b*_ + 3*T*_*mp*_ + 1*T*_*e*_ + 5*T*_*h*_	2*T*_*b*_ + 4*T*_*mp*_ + 2*T*_*e*_ + 6*T*_*h*_	49.421 ms
Wazid et al. (2017)	4*T*_*mp*_ + 2*T*_*eca*_ + 5*T*_*h*_	–	2*T*_*mp*_ + 1*T*_*fe*_ + 8*T*_*h*_	6*T*_*mp*_ + 2*T*_*eca*_ + 1*T*_*fe*_ + 13*T*_*h*_	31.088 ms
Odelu et al. (2016)	3*T*_*mp*_ + 1*T*_*e*_ + 6*T*_*h*_	–	2*T*_*mp*_ + 2*T*_*b*_ + 1*T*_*e*_ + 6*T*_*h*_	5*T*_*mp*_ + 2*T*_*e*_ + 1*T*_*b*_ + 12*T*_*h*_	40.882 ms
Gope & Sikdar (2018)	1*T*_*fe*_ + 5*T*_*h*_ + 1*T*_*PUF*_	–	1*T*_*fe*_ + 6*T*_*h*_	2*T*_*fe*_ + 11*T*_*h*_ + 1*T*_*PUF*_	4.766 ms
Kumar et al. (2018)	3*T*_*mp*_ + 2*T*_*s*_ + 4*T*_*h*_	3*T*_*mp*_ + 2*T*_*s*_ + 5*T*_*h*_	–	6*T*_*mp*_ + 4*T*_*s*_ + 9*T*_*h*_	35.875 ms
Zhang et al. (2019)	1*T*_*s*_ + 7*T*_*h*_	–	3*T*_*s*_ + 9*T*_*h*_	4*T*_*s*_ + 16*T*_*h*_	0.483 ms
Ma et al. (2019)	1*T*_*mp*_ + 2*T*_*s*_ + 7*T*_*h*_	–	1*T*_*mp*_ + 4*T*_*s*_ + 14*T*_*h*_	2*T*_*mp*_ + 6*T*_*s*_ + 21*T*_*h*_	9.158 ms
Wu et al. (2019)	3*T*_*e*_ + *T*_*mp*_ + 4*T*_*h*_	5*T*_*e*_ + *T*_*mp*_ + 4*T*_*h*_	–	8*T*_*e*_ + 2*T*_*mp*_ + 8*T*_*h*_	74.888 ms
Li et al. (2019)	2*T*_*e*_ + *T*_*s*_ + 4*T*_*h*_	3*T*_*e*_ + *T*_*mp*_ + 4*T*_*h*_	–	5*T*_*e*_ + *T*_*s*_ + *T*_*mp*_ + 4*T*_*h*_	46.198 ms
Khan, Kumar & Ahmad (2019)	2*T*_*mp*_ + 1*T*_*fe*_ + 4*T*_*h*_	–	2*T*_*mp*_ + 3*T*_*h*_	4*T*_*mp*_ + 1*T*_*fe*_ + 7*T*_*h*_	20.417 ms
Proposed	2*T*_*mp*_ + 12*T*_*h*_	1*T*_*mp*_ + 12*T*_*h*_	1*T*_*mp*_ + 7*T*_*h*_	4*T*_*mp*_ + 31*T*_*h*_	21.001 ms

Comparing the proposed scheme’s results with the other schemes presented in Table 8, we can observe that the proposed scheme’s computational cost and estimated execution time is higher than Gope & Sikdar (2018), Zhang et al. (2019), Ma et al. (2019), Khan, Kumar & Ahmad (2019) schemes and lesser than all other schemes. Unlike the proposed scheme, Gope & Sikdar (2018), Zhang et al. (2019), Ma et al. (2019) schemes will not mutually authenticates every participants involved in the communication. There are some architectural limitations where the scheme can be applied only when the smart meter is directly communicates with the smart grid. As discussed in “Introduction”, smart grid topology is split into several networks which contains several smart meters, NAN gateways, and SG. Therefore, the schemes of Gope & Sikdar (2018), Zhang et al. (2019), Ma et al. (2019) are not efficient for hierarchical model of smart grid communications. The estimated execution time of Khan, Kumar & Ahmad’s (2019) scheme also less than the proposed scheme. But the khan2019elliptic scheme was limited to authenticate smart meters and NAN gateway only. The proposed scheme computation cost and estimated execution time include the mutual authentication of a smart meter, NAN gateway, and smart grid. Also, the authentication of the proposed scheme has been achieved without involvement of any third party. Therefore the computation cost looks higher than the schemes of Gope & Sikdar (2018), Zhang et al. (2019), Ma et al. (2019), Khan, Kumar & Ahmad (2019). However, the proposed scheme overcomes all the security barriers presented in “Functional Analysis”, and achieves fully distributed multistage authentication.

### Communication cost

**Figure 5:**
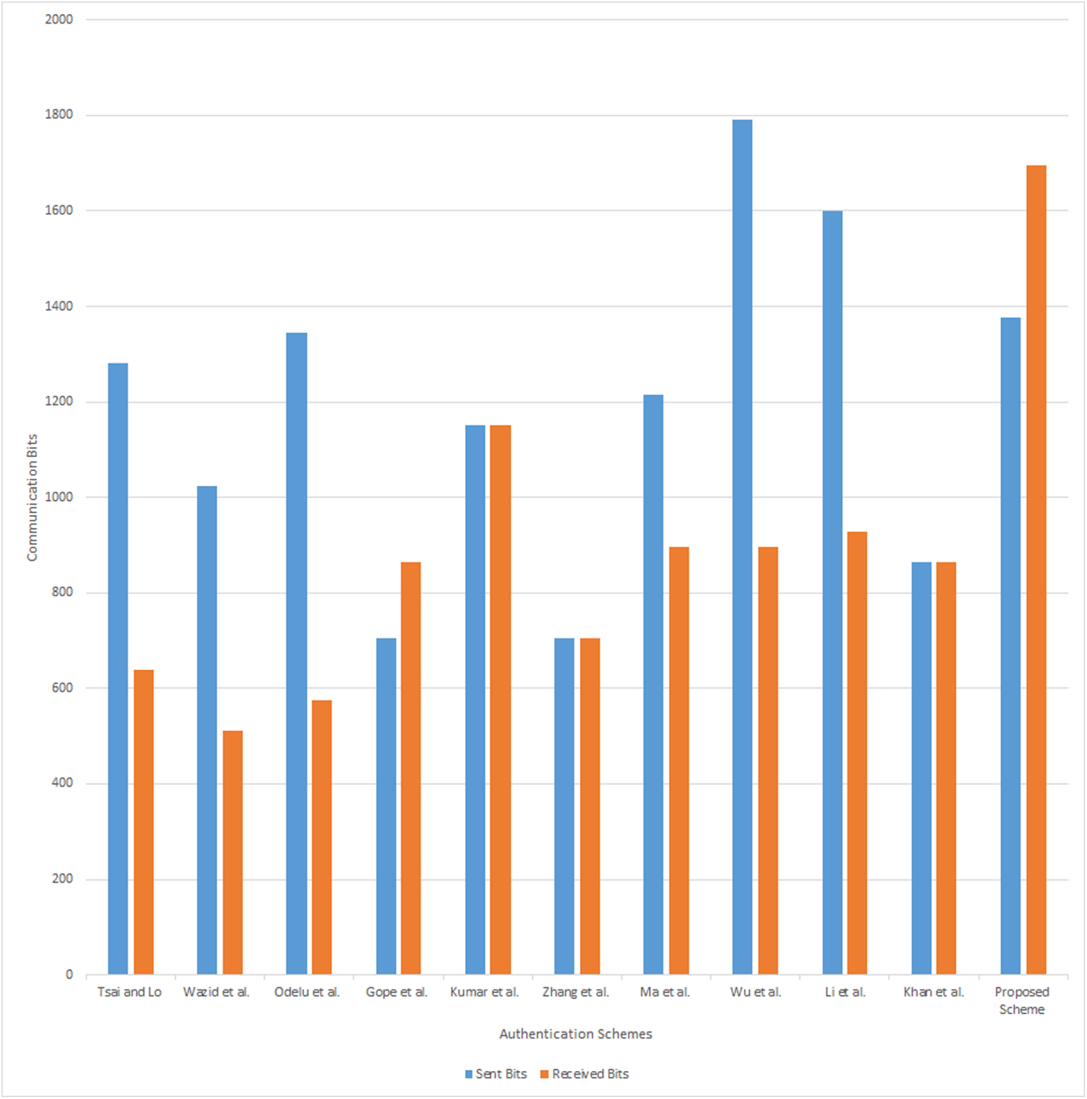
Communication analysis.

The communication cost of the proposed scheme is compared with the Tsai & Lo (2015), Wazid et al. (2017), Odelu et al. (2016), Gope & Sikdar (2018), Kumar et al. (2018), Zhang et al. (2019), Ma et al. (2019), Wu et al. (2019), Li et al. (2019) and Khan, Kumar & Ahmad (2019) schemes and presented in Table 9. It includes the cost of the communication parameters, transmitted in one complete session of the authentication phase. For consistency purpose, we assume that the length of the identity *ID*_*i*_ and random number is 128 bits, the output size of hash functions *H*_0_ (.), *H*_1_ (), and *H*_2_ () is 160 bits, size of an elliptic curve point is 320 bits, the block size of symmetric encryption/decryption is 256 bits, size of bilinear pairing is ${G_1} \to 320$ bits, ${G_2} \to 512$ bits and a Timestamp is 32 bits. The authentication phase of the proposed scheme requires 320 + 160 + 160 + 32 = 672 bits, 160 + 160 + 160 + 160 + 32 + 32 = 704 bits, 160 + 320 + 160 + 32 = 672 bits, and 320 + 160 + 160 + 160 + 160 + 32 + 32 = 1074 bits, for the messages {*C*_*sm*_, *CID*_*i*_, *C*_1_, *T*_1_}, {*CID*_*i*_, *C*_2_, *C*_*nan*_, *RID*_*s*_, *T*_1_, *T*_2_}, {*C*_3_, *C*_*s*_, *T*_3_}, and {*C*_*s*_, *T*_3_, *C*_4_, *C*_*m*_, *T*_4_, *RID*_*m*_}. Hence, the total communication cost required for the proposed scheme to achieve the one session is 3072 bits. The communication cost analysis result is presented in Fig. 5.

**Table 9:** Communication cost analysis.

Schemes	Send		Receive		Total	
	Comm. bits	Energy cost (*μJ*)	Comm. bits	Energy cost (*μJ*)	Comm. bits	Energy cost (*μJ*)
Tsai & Lo (2015)	1,280	921.6	640	518.4	1,920	1,440
Wazid et al. (2017)	1,024	737.28	512	414.72	1,536	1,152
Odelu et al. (2016)	1,344	967.68	576	466.56	1,920	1,434.24
Gope & Sikdar (2018)	704	506.88	864	699.84	1,568	1,206.72
Kumar et al. (2018)	1,152	829.44	1,152	933.12	2,304	1,762.56
Zhang et al. (2019)	704	506.88	704	570.24	1,408	1,077.12
Ma et al. (2019)	1,216	875.52	896	725.76	2,112	1,601.28
Wu et al. (2019)	1,792	1290.24	896	725.76	2,688	2,016
Li et al. (2019)	1,600	1152	928	751.68	2,528	1,903.68
Khan, Kumar & Ahmad (2019)	864	622.08	864	699.84	1,728	1,321.92
Proposed Scheme	1,376	990.72	1,696	1,373.76	3,072	2,364.48

**Figure 6:**
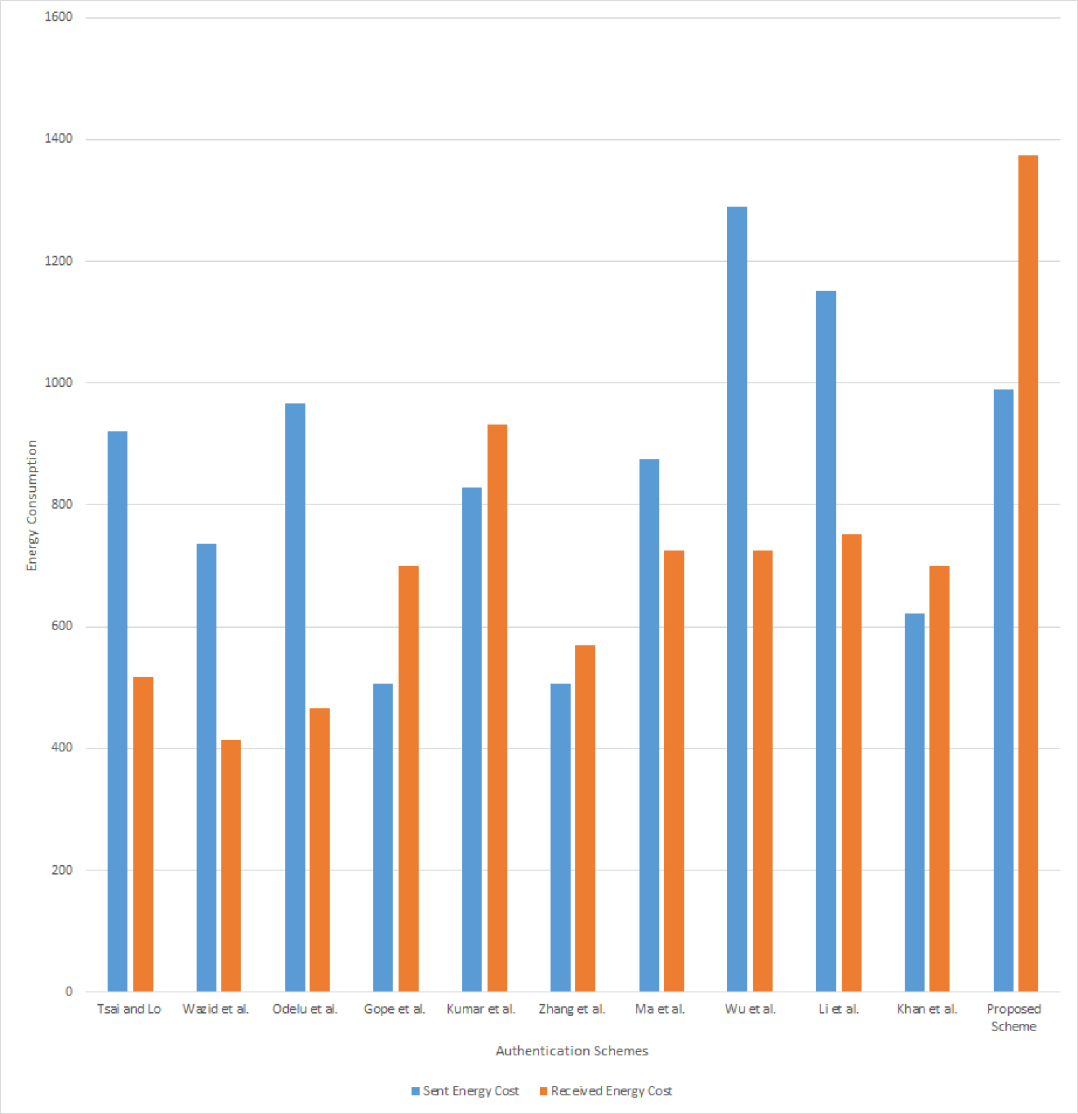
Energy consumption in communication.

We have also calculated the energy cost of the proposed scheme and compared the result with other schemes. The energy cost gives the expected energy required to communicate the data in the communication channel. We have used De Meulenaer et al. (2008) model to compute the energy cost. According to this model, the energy required to send and receive 1 bit of data is 0.72 *μJ* and 0.81 *μJ*, respectively. The communication cost of the sent message is 1,376 bits and received message is 1,696 bits, respectively. Hence, the energy cost of the proposed scheme for send and receive messages are 1,376 × 0.72 = 990.72 *μJ* and 1,696 × 0.81 = 1,373.76 *μJ*. The total expected energy cost of the proposed scheme is 2,364.48 *μJ*. The result of energy cost analysis has been presented in Table 9 and also shown in Fig. 6.

According to the analysis result presented in Table 9, the communication and energy costs of the proposed scheme are higher than all other schemes. Unlike other schemes the proposed scheme cost includes the communication done between smart meter, NAN gateway, and smart grid during the authentication. In Table 9, Tsai & Lo (2015), Wazid et al. (2017), Odelu et al. (2016), Gope & Sikdar (2018), Zhang et al. (2019), Ma et al. (2019), and Khan, Kumar & Ahmad (2019) schemes, have no communication with NAN gateway during authentication. Therefore, during communication cost comparison, if we neglect the proposed scheme communication messages done with NAN gateway, the total communication cost of the messages {*C*_*sm*_, *CID*_*i*_, *C*_1_, *T*_1_}, {*C*_3_, *C*_*s*_, *T*_3_} is 1,344 bits and the energy cost is 1,028.16 *μJ* which is less than other schemes. Similarly, Kumar et al. (2018), Wu et al. (2019), and Li et al. (2019) schemes have no communication between NAN gateway and smart grid, we neglect those communication done in the proposed scheme to do the comparison. Therefore the total communication cost of the messages {*C*_*sm*_, *CID*_*i*_, *C*_1_, *T*_1_}, {*C*_*s*_, *T*_3_, *C*_4_, *C*_*m*_, *T*_4_, *RID*_*m*_} is 1,746 bits and the energy cost is 1,353.78 *μJ* which is lesser than other compared schemes. Hence, we claim that the proposed scheme is efficient than other schemes and robust in the multistage architecture.

### Functional analysis

Table 10 presents the functional analysis of the proposed scheme done with other schemes. The features and the functionalities represented in Table 8 are as follows: F1—Multistage authentication, F2—Provide perfect forward secrecy, F3—Prevents replay attack, F4— Prevents insider attack, F5—Prevents man in middle attack, F6—Prevents impersonate attack, F7—Provide mutual authentication, F8— Provide smart meter credentials privacy, F9—Provides session key security. From Table 10, it is clear that the proposed scheme meets all the functional requirements. It also achieves multistage authentication where one smart grid can authenticate the smart meter through the NAN gateway.

**Table 10:** Functional analysis.

Schemes	F1	F2	F3	F4	F5	F6	F7	F8	F9
Tsai & Lo (2015)	×	*✓*	×	×	*✓*	*✓*	*✓*	×	×
Wazid et al. (2017)	×	×	*✓*	×	*✓*	*✓*	*✓*	*✓*	*✓*
Odelu et al. (2016)	×	*✓*	×	*✓*	*✓*	*✓*	*✓*	*✓*	*✓*
Gope & Sikdar (2018)	×	*✓*	×	*✓*	*✓*	*✓*	×	*✓*	*✓*
Kumar et al. (2018)	×	*✓*	*✓*	*✓*	*✓*	*✓*	*✓*	×	*✓*
Zhang et al. (2019)	×	*✓*	×	×	*✓*	*✓*	*✓*	×	*✓*
Ma et al. (2019)	×	*✓*	×	*✓*	×	*✓*	*✓*	×	*✓*
Wu et al. (2019)	×	*✓*	*✓*	*✓*	*✓*	*✓*	*✓*	×	*✓*
Li et al. (2019)	×	*✓*	*✓*	*✓*	×	*✓*	*✓*	×	*✓*
Khan, Kumar & Ahmad (2019)	×	×	*✓*	*✓*	×	*✓*	*✓*	*✓*	*✓*
Proposed	*✓*	*✓*	*✓*	*✓*	*✓*	*✓*	*✓*	*✓*	*✓*

## Conclusions

The security of smart meter communication is critical for reliable, efficient and stable operation of a contemporary smart grid. This paper addresses the security issues by considering a novel authentication scheme for smart meter communication with the energy control center. The proposed method is based on a novel formulation, where the authentication scheme is dynamic, multi-stage and distributed in nature. The formal proves presented in this paper validate that the proposed model can establish a secure authentication path between a smart meter, NAN gateway, and a SCADA energy center in a distributed manner. We also verified the effectiveness of the proposed authentication scheme against adversarial attacks using a simulation tool AVISPA. Considering the computation, communication and functional costs, this article also presented the efficiency analysis of the proposed authentication scheme. Based on comparative analyses, we can conclude that the proposed scheme is robust and secure as compared to the existing related schemes. Hence, the proposed authentication scheme has the potential to secure smart meter communication between the energy consumers and utility control centers to foster an attack resilient sustainable energy grid.

## Appendix

**Figure 7:**
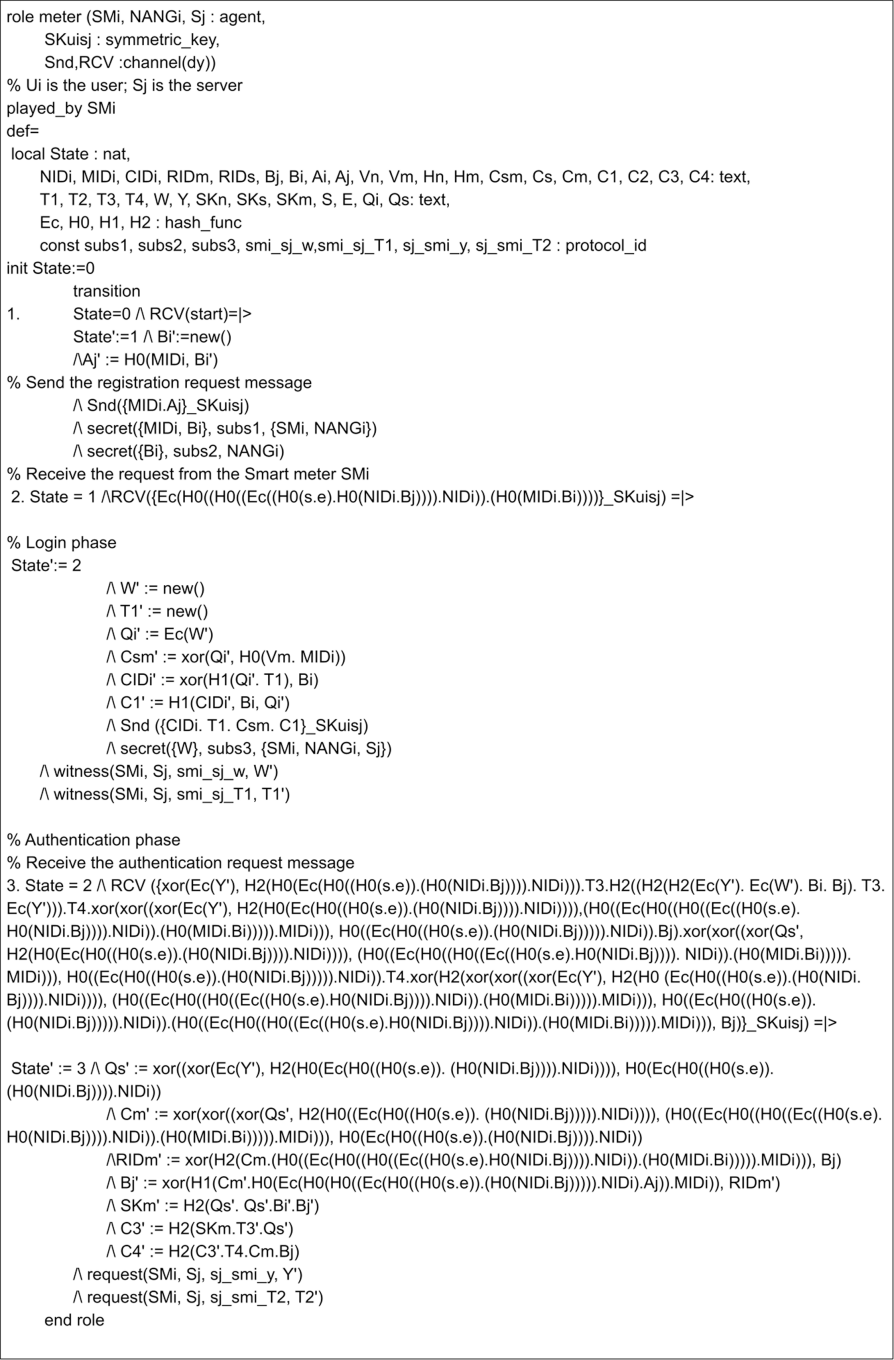
Role specification for the SMi of the proposed scheme.

**Figure 8:**
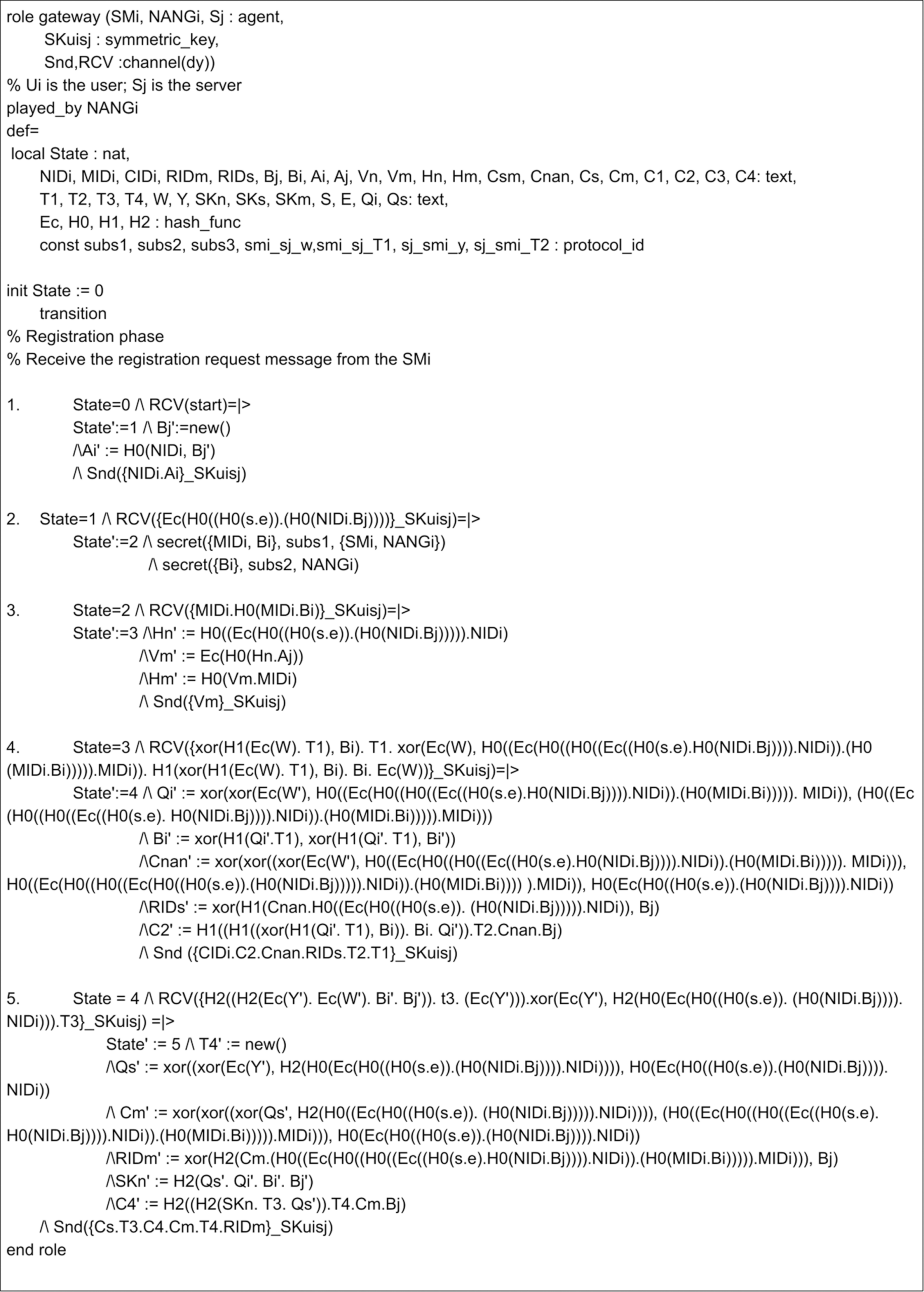
Role specification for the NANGi of the proposed scheme.

**Figure 9:**
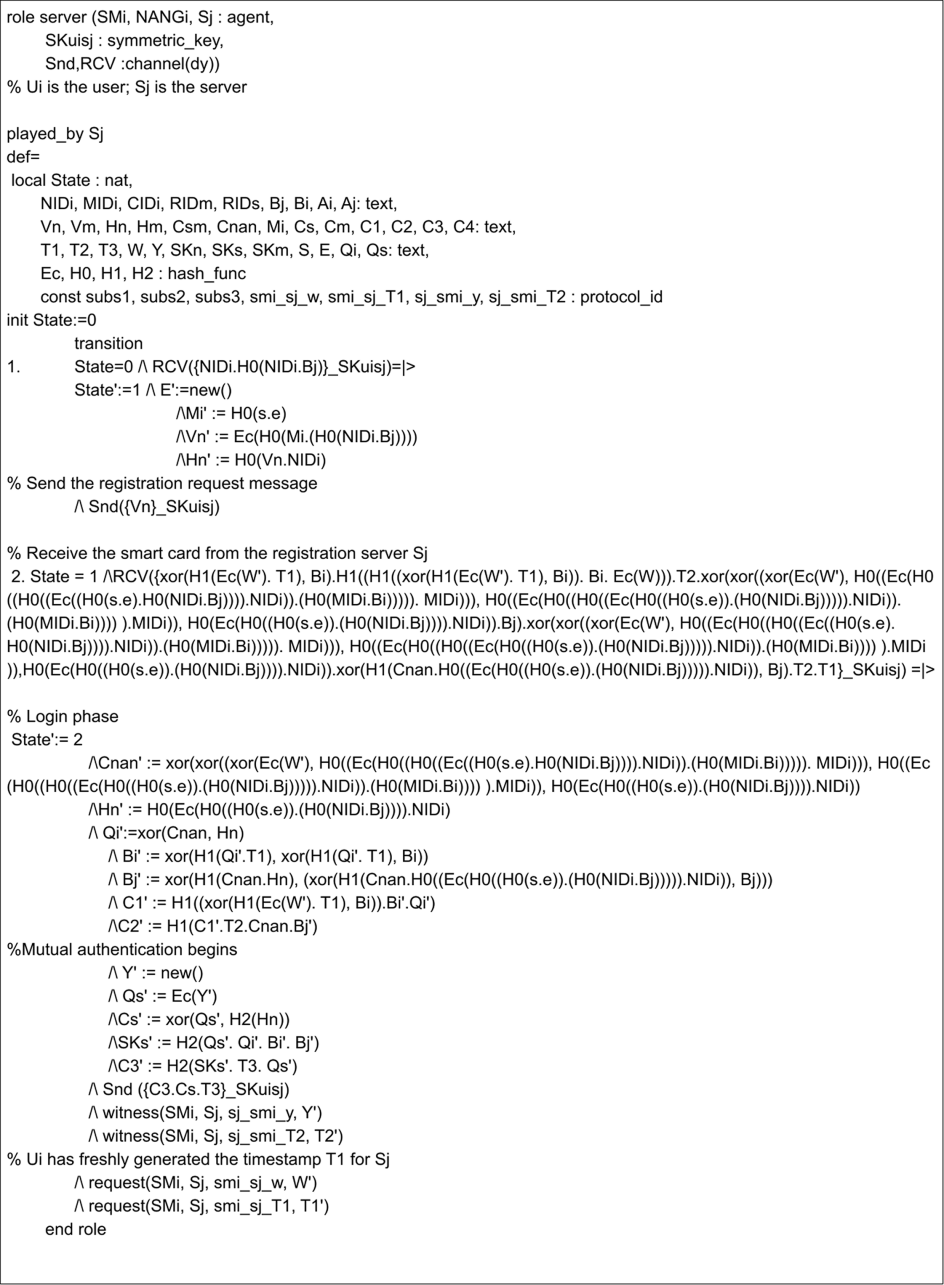
Role specification for the SGi of the proposed scheme.

The implementation of the proposed scheme in AVISPA includes the registration phase and the login and authentication phase. Here, three leading roles are named as meter, gateway, and server, represented as *SM*_*i*_, *NANG*_*i*_ and *S*_*j*_. The role specification of the meter is presented in Fig. 7. Here, the process begins by receiving a start signal. The gateway and server roles implementations are given in Figs. 8 and 9. *SM*_*i*_, *NANG*_*i*_ and *S*_*j*_ use symmetric key SKuisj for the communication in the channel. The send and receive channels required for communication between meter, gateway, and server are represented by Snd() and Rcv() functions.

**Figure 10:**
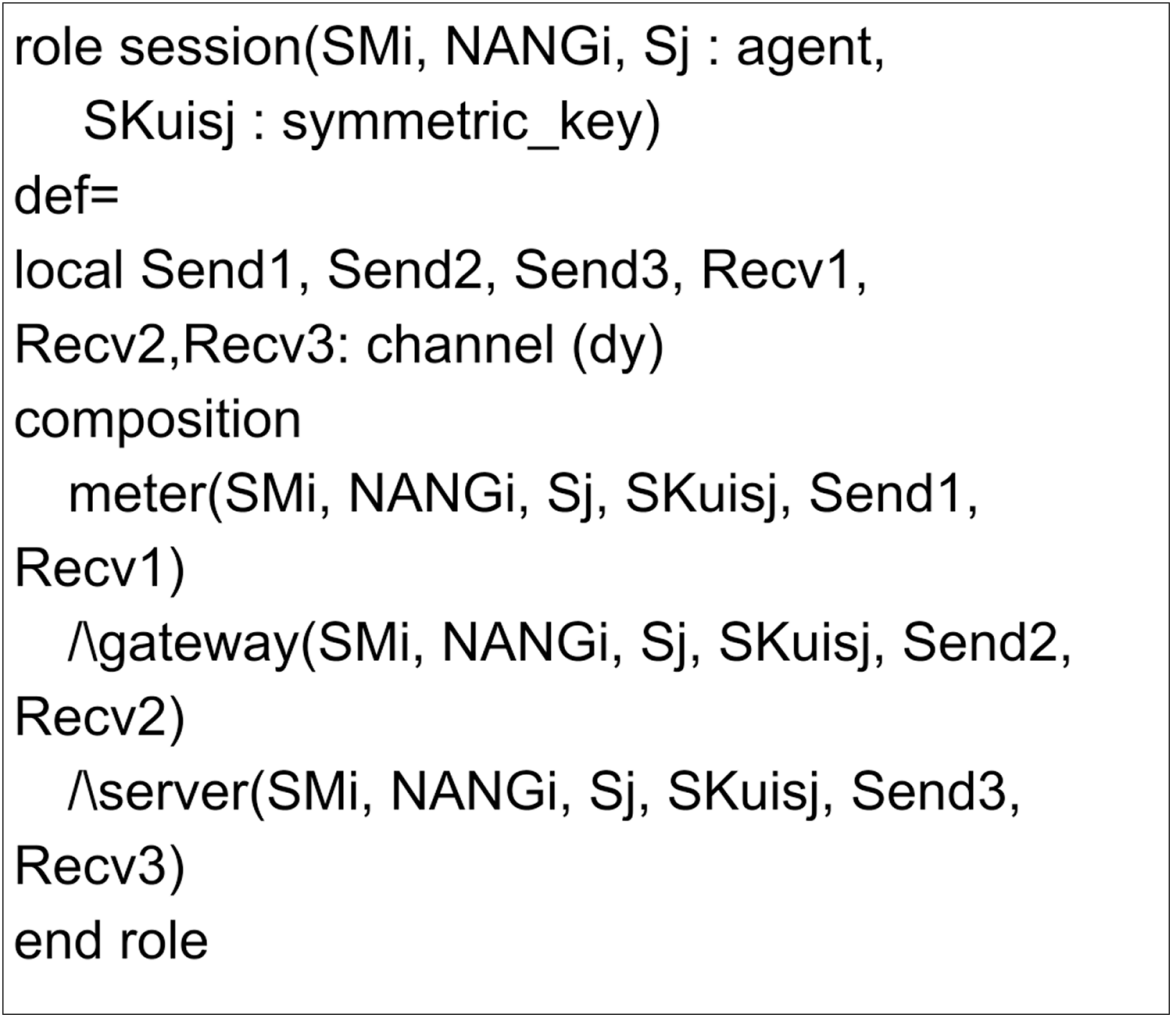
Role specification for the session of the proposed scheme.

**Figure 11:**
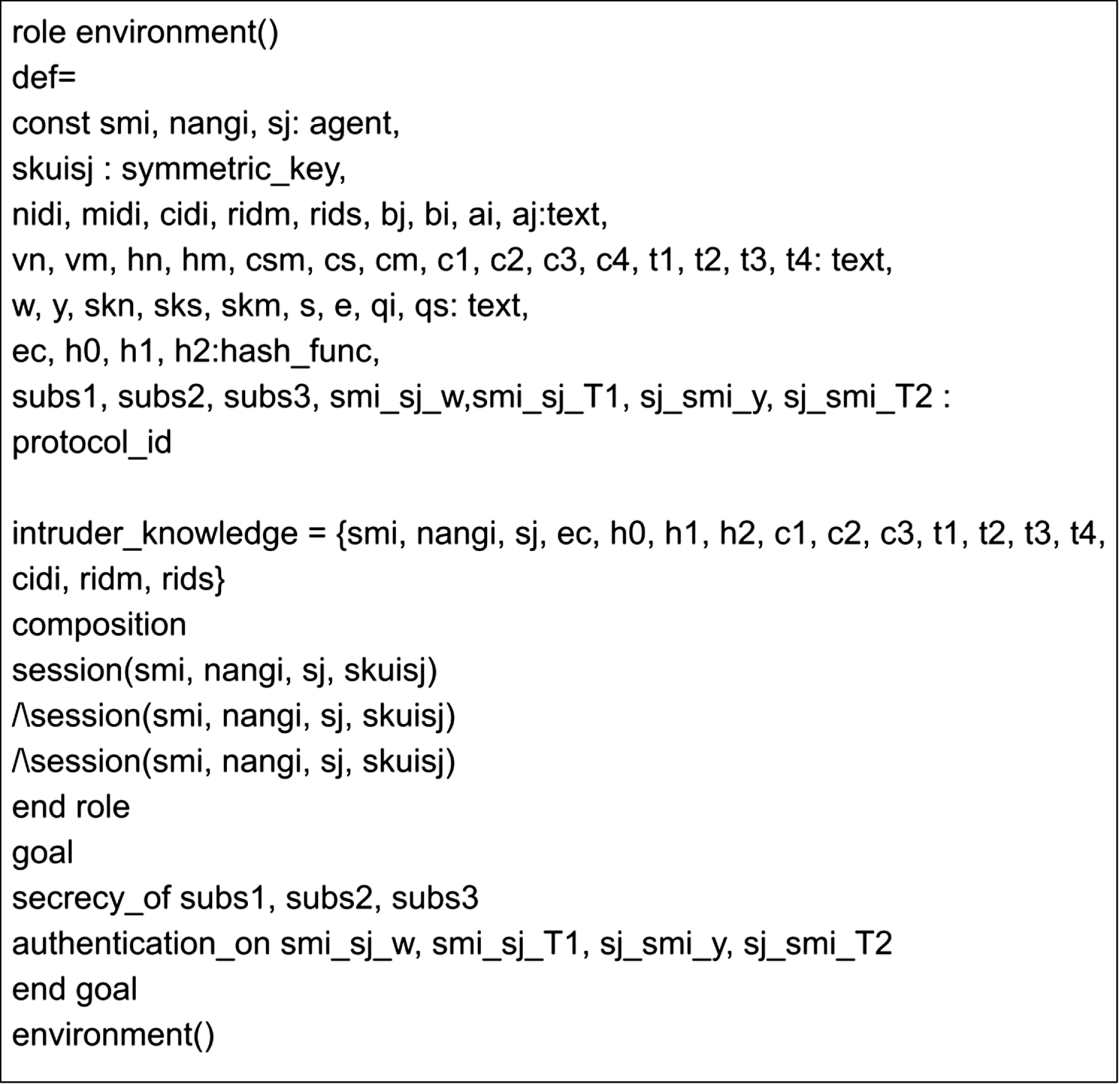
Role specification for the goal and environment of the proposed scheme.

Figures 10 and 11 present the session and environment roles. The session role includes the primary roles for composition and the channels of all roles involved in communication. The environment role specifies the global constants and sessions for an adversary to play as legitimate user roles. It also defines the goals of the proposed scheme.

## Supplemental Information

10.7717/peerj-cs.643/supp-1 Supplemental Information 1 Raw data and HLPSL code used for the implementation.

## Additional Information and Declarations

### Data Availability

The following information was supplied regarding data availability: The code is available in the Supplemental Files.
